# Supplementary material for: Addressing pandemic-wide systematic errors in the SARS-CoV-2 phylogeny
Source: Nat Methods. 2026 Feb 9;23(3):653–62. doi: 10.1038/s41592-025-02947-1 (PMC12982125; doi:10.1038/s41592-025-02947-1)
Supplement: Supplementary file 1 — Supplementary Text and Figs. 1–9. [file 41592_2025_2947_MOESM1_ESM.pdf]

nature methods

Supplementary information https://doi.org/10.1038/s41592-025-02947-1

Addressing pandemic-wide systematic errors in the SARS-CoV-2 phylogeny

In the format provided by the authors and unedited

Contents

1 Reference calls with zero read depth **2**

2 Pandemic timeline **3**

3 Primer scheme identification validation **4**

4 Run time and memory **10**

5 Assembly and evaluation of the global data **11**

6 Indel calls **12**

7 Reversions **13**

8 Improved accuracy of lineage growth rate estimate **14**

9 Measuring uncertainty in the global tree **17**

10 Impact on evolutionary and epidemiological analysis **19**

11 mpox **22**

12 Geographical distribution of samples **25**

13 Methods **27**

14 IMSSC2 Laboratory Network Consortium members **35**

1 Reference calls with zero read depth

We used all consensus sequences from the "intersection" data set, which comprises all samples that have both a Viridian and GenBank assembly available. It is defined in the main text in the section "Assembly and evaluation of the global data".

The read depths were obtained from running Viridian with the option `--force_consensus`. The full Viridian methods are explained in the main text. The main point here to understand this analysis, is that it means Viridian takes the input assembly (in this case, a Viridian or GenBank consensus sequence), and outputs per-base QC information of the assembly, using the pileup of the reads. This QC information is generated after per-amplicon sampling of reads to a mean of 1000X depth across each amplicon. However, if an amplicon has mean depth less than 1000X, then all reads are kept for that amplicon, so that we do not expect to call false-positive zero-depth regions for this analysis. The primer portions of reads do not count towards the pileup read depths. Using these results, for each sample we identified regions of at least 50bp in length that had zero read depth but were identical to the reference genome. Such regions should comprise of Ns, since there is no evidence from the reads to support a call of A, C, G, or T.

A summary of the prevalence of reference-called but zero read depth regions is shown in Supplementary Figure 1, calculated as follows. For each sample, a zero depth region with all reference calls, say ranging from positions A to B, would mean incrementing the counter at all positions from A to B inclusive. This was calculated for each of Viridian and GenBank, giving an overall summary of these regions across all samples.

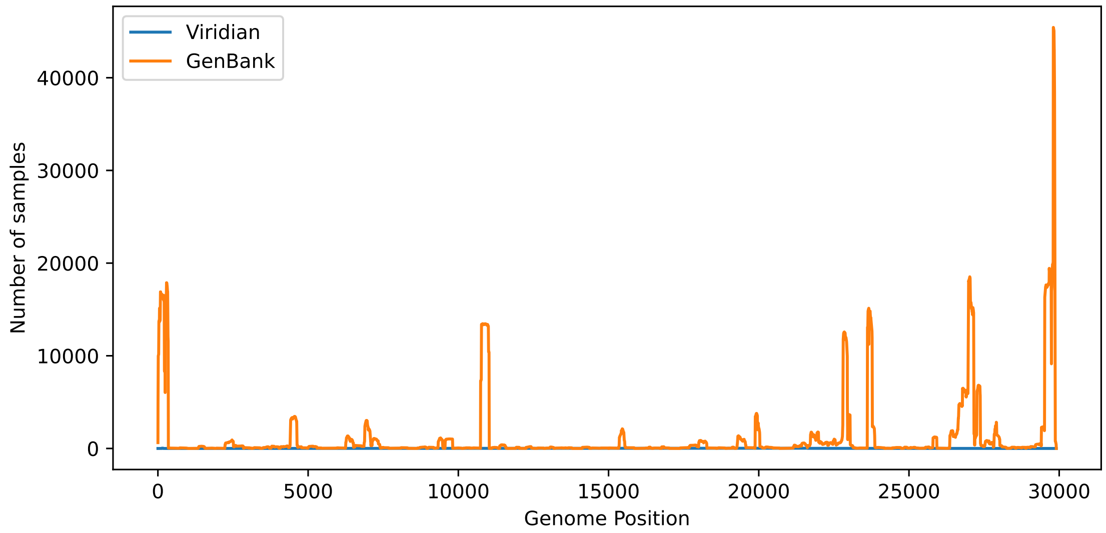

Figure 1: Prevalence of consensus sequence calls that are the same as the reference, but have zero read depth to support them.

2 Pandemic timeline

The same timeline as in the main Figure 1 is shown in Supplementary Figure 2, but with plots added showing the number of masked sites and nodes in the global phylogenetic tree of SARS-CoV-2.

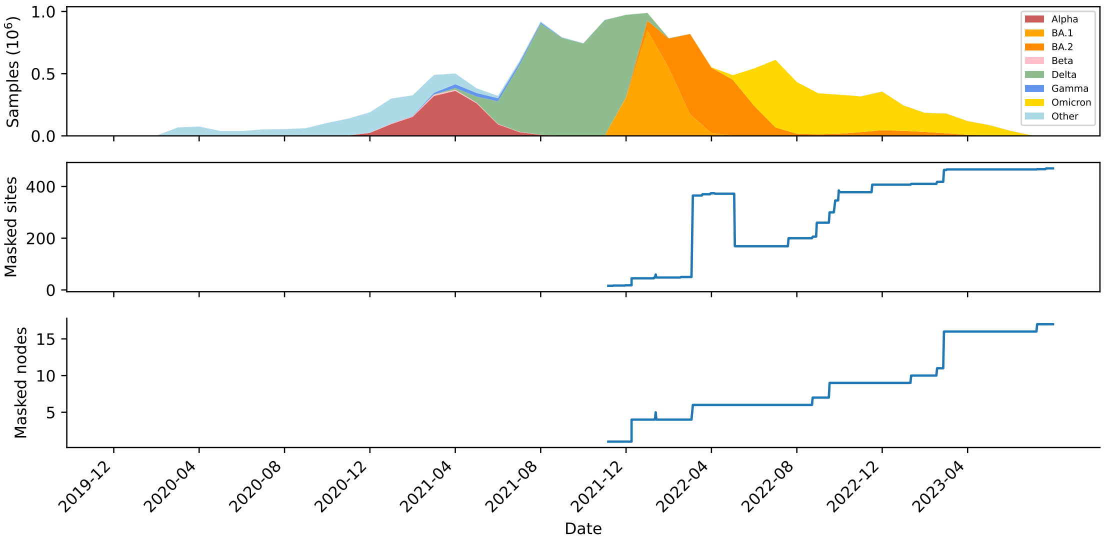

Figure 2: Timeline of the SARS-CoV-2 pandemic from December 2019 to July 2023, plus the number of masked sites and nodes in the SARS-CoV-2 global phylogenetic tree.

3 Primer scheme identification validation

Example scheme score plots output by Viridian for a very clean Illumina sample (ERR9362110) and an Illumina sample with fragmented reads (ERR8959211) are shown in Supplementary Figure 3. These are from the truth dataset, and are typical of those samples: the ARTIC version 3 Ilumina reads (eg ERR8959211) are fragmented due to tagmentation during library preparation, whereas the ARTIC version 4 reads are not. Artemis screenshots of the reads from these runs are given in Supplementary Figure 4, showing the difference between the two runs.

ERR9362110 was sequenced using ARTIC scheme version 3, which Viridian scored at 4920. The other scores ranged from -278 to 632. ERR8959211 was sequenced using ARTIC scheme version 4.1, which Viridian scored at 2372. The other scores ranged from -126 to 504. The comparatively lower score of 2372 is a result of the fragmented reads, but is still 4.7 times greater than the second best score. This shows that Viridian successfully calls the scheme even when the reads within each amplicon are fragmented.

The accessions of the manually checked runs that were discordant between the ARTIC primer scheme version in the INSDC metadata and the Viridian call were:

- Illumina, INSDC=3, Viridian=4: ERR7207071, ERR7687763, ERR7696315, ERR7704807, ERR7713199
- Illumina, INSDC=4, Viridian=3: ERR6435020, ERR7202077, ERR7306912, ERR8190486, ERR8228569
- Nanopore, INSDC=3, Viridian=4: ERR5226357, ERR8202943, ERR8218048, ERR8235241, ERR8250042
- Nanopore, INSDC=4, Viridian=3: ERR5226357, ERR5401980, ERR5516251, ERR6114066, ERR6207127.

All Nanopore runs followed the same pattern: reads mapped at positions corresponding exactly to complete amplicons, and all matched the scheme version called by Viridian. Artemis screenshots of Nanopore run ERR5226357 are shown in Supplementary Figure 5. The Illumina reads were fragmented, but with enough signal to determine that the Viridian call was correct in 9 of the 10 runs, and the remaining run ERR8228569 was inconclusive. Artemis screenshots of Illumina run ERR7704807 are shown in Supplementary Figure 6, which is typical of the 9 runs whose scheme was manually identified. The inconclusive run ERR8228569 is shown in figure 7.

The screenshots focus on the last ∼10kb of the genome, since this is where the amplicons differ most between the two scheme versions. Reads were randomly sampled using SAMtools with the `-s` option before viewing to aid visualisation, since the full depth results in stacks of reads being too high for the viewing window and therefore not visible.

(a) **(b)**

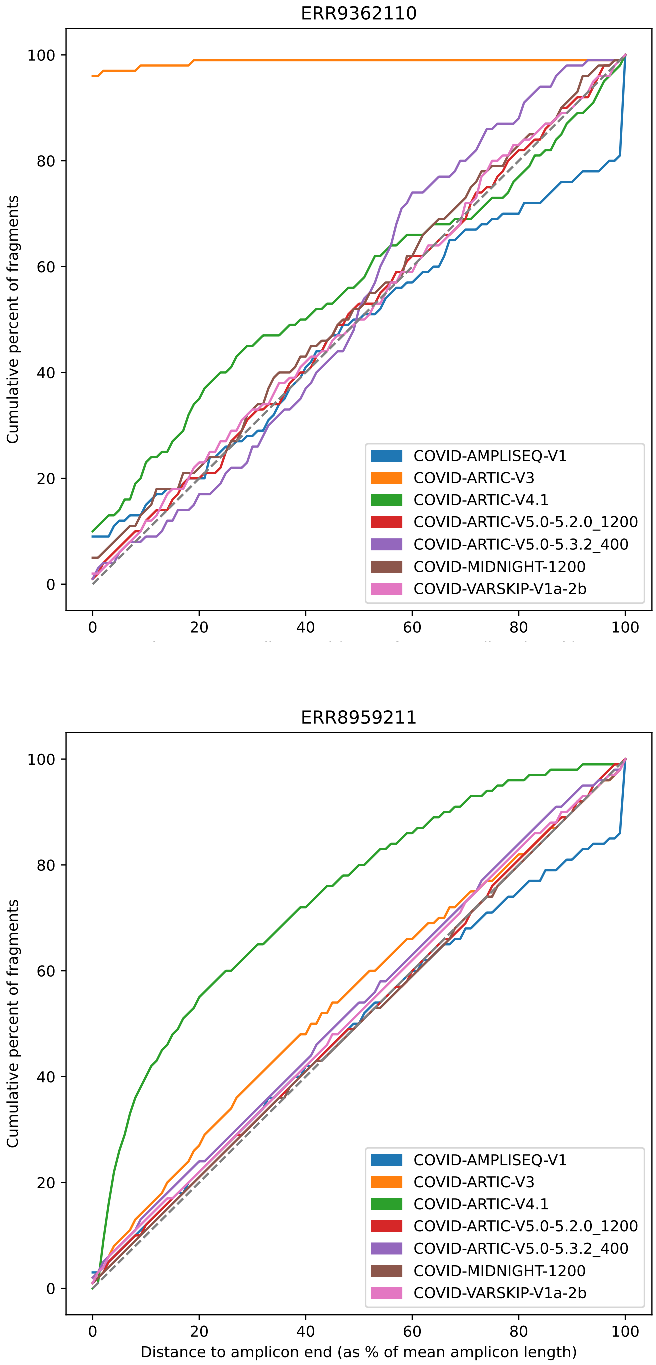

Figure 3: Scheme identification plot output by Viridian for Illumina runs (a) ERR9362110 and (b) ERR8959211.

(a)

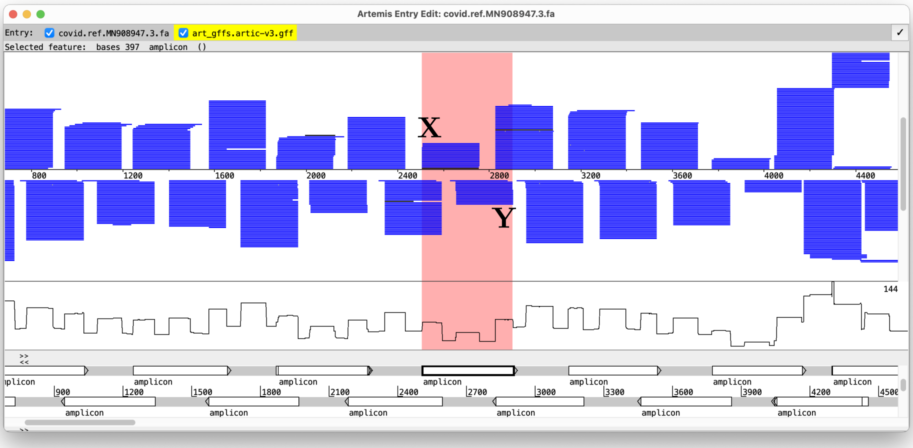

(b)

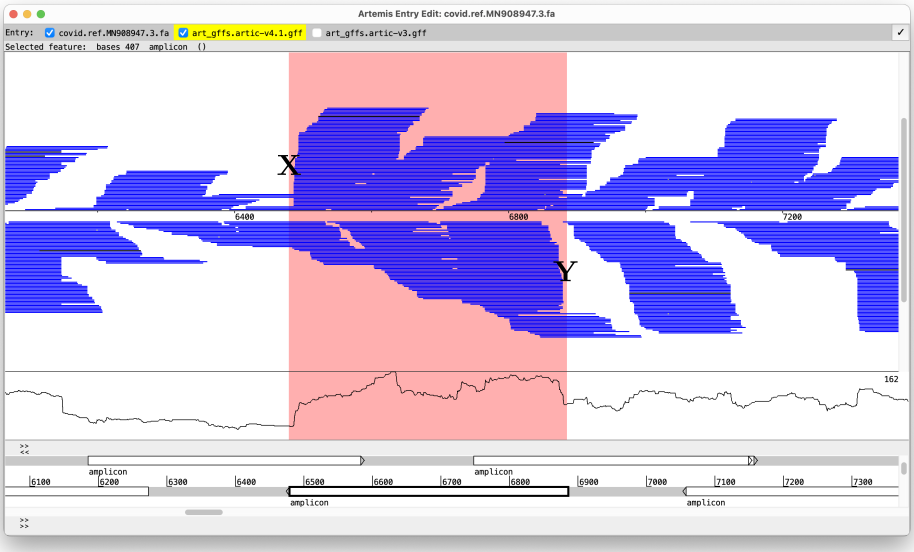

Figure 4: Artemis screenshots showing reads mapped to the SARS-CoV-2 reference genome: (a) ERR9362110; (b) ERR8959211. Reads are shown using the "strand stack" view, where the upper reads are those that map in the forwards orientation, and the lower reads are those mapped in the reverse direction (flag 16 in the BAM file). The line plot below the reads shows the read depth across the genome. Since amplicons overlap, they are shown as annotated alternating between the forward and reverse strands. This is to aid visualization and the apparent strand/direction of each amplicon is not relevant. It is their positions that is important. An amplicon shown on top of another amplicon is where there are alternative primers for the same amplicon, for example (a) at position ~4,400. One amplicon is highlighted in each screenshot to illustrate how the ends of mapped reads match. We are looking for reads mapped to the forwards strand with left ends matching the amplicon start (marked with an X), and reads mapped to the reverse strand with right ends matching the amplicon end (marked with a Y): in (a) they match perfectly, in (b) there is enough of a signal to see that the reads match, but is less clear.

(a)

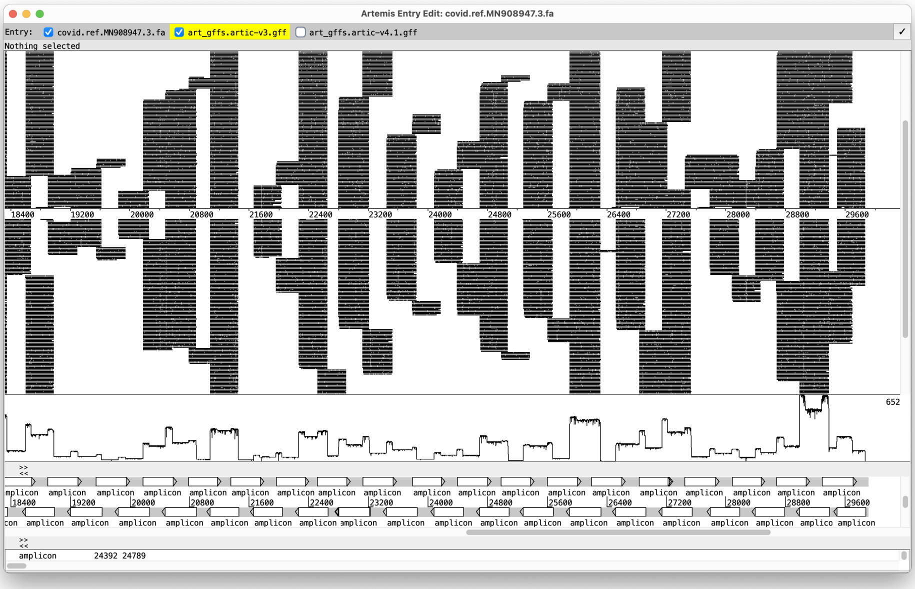

(b)

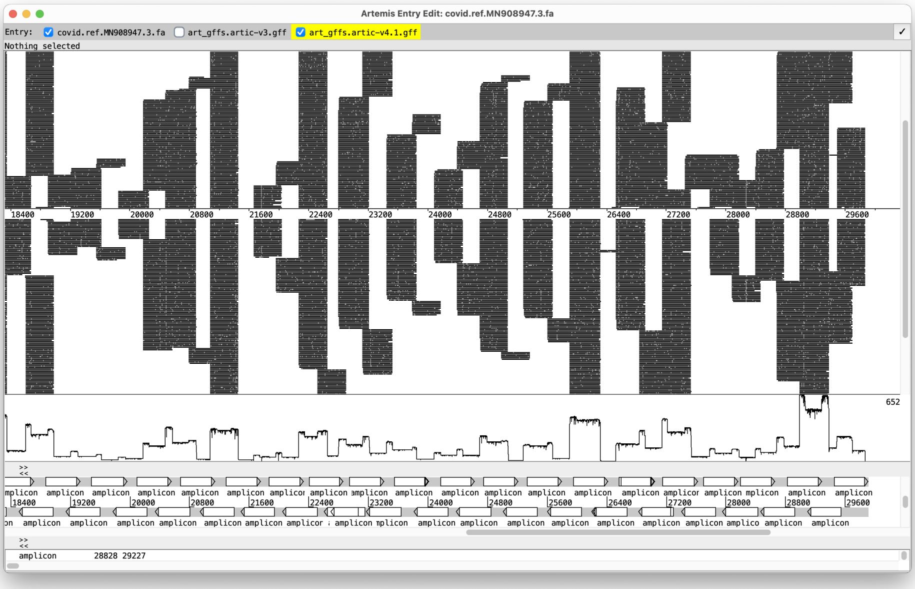

Figure 5: Artemis screenshots showing reads from Nanopore run ERR5226357 mapped to the SARS-CoV-2 reference genome. The screenshots are identical, except for the lower track showing the amplicons from ARTIC primer scheme version 3 in (a) and version 4 in (b). Reads are shown using the "strand stack" view, where the upper reads are those that map in the forwards orientation, and the lower reads are those mapped in the reverse direction (flag 16 in the BAM file). The line plot below the reads shows the read depth across the genome. Since amplicons overlap, they are shown as annotated alternating between the forward and reverse strands. This is to aid visualization and the apparent strand/direction of each amplicon is not relevant. It is their positions that is important. An amplicon shown on top of another amplicon is where there are alternative primers for the same amplicon, for example (b) at position ~22,700. The reads match perfectly to scheme version 3.

(a)

(b)

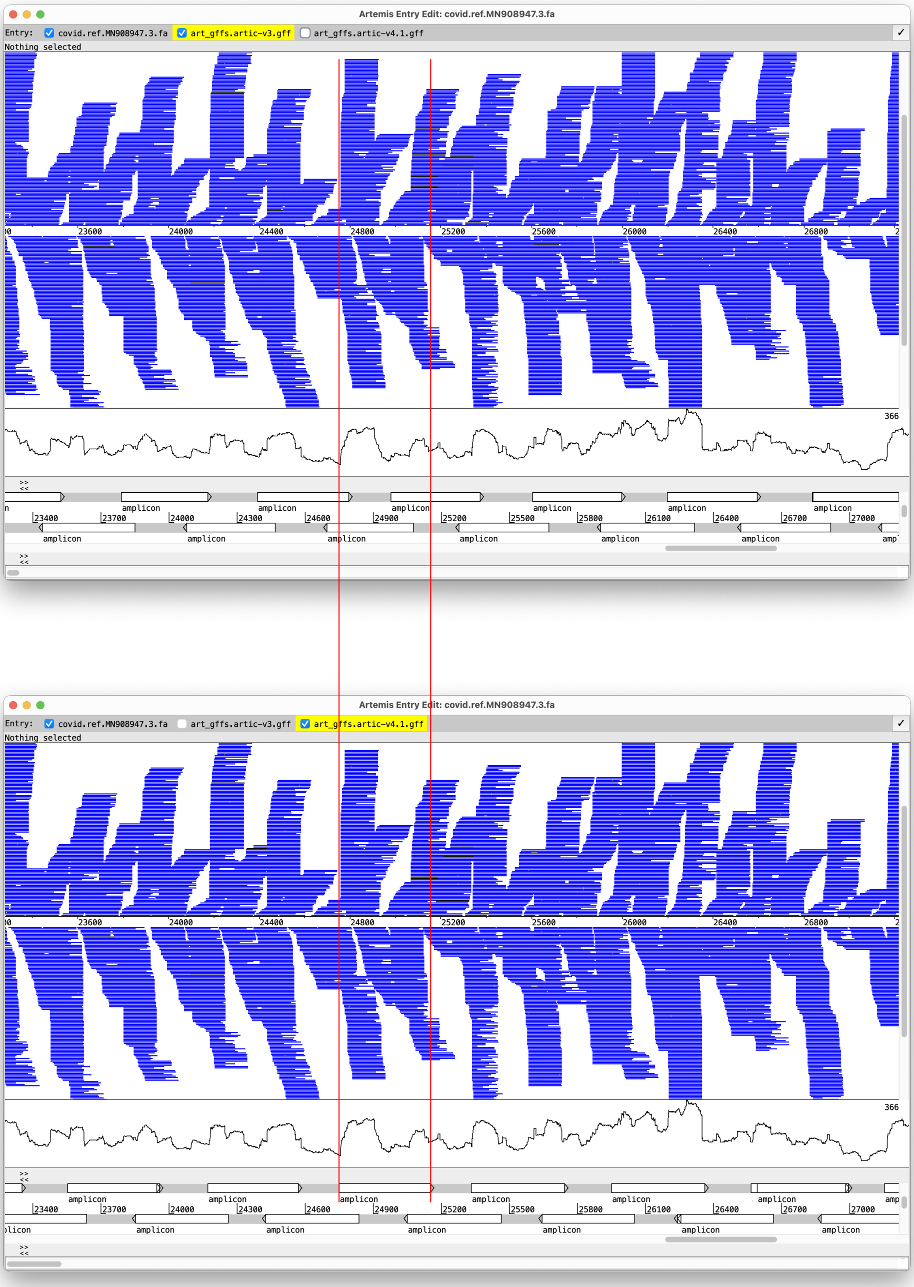

Figure 6: Artemis screenshots showing reads from Illumina run ERR7704807 mapped to the SARS-CoV-2 reference genome. (a) ARTIC amplicon scheme version 3 is annotated. (b) ARTIC amplicon scheme version 4 is annotated. See the legend of Supplementary Figure 6 for an explanation of the visualisation details. The reads best match scheme version 4: large increases/decreases in read depth match the start/end of amplicons, and there are peaks of greater read depth where adjacent amplicons overlap. See for example the amplicon marked by the two vertical red lines.

(a)

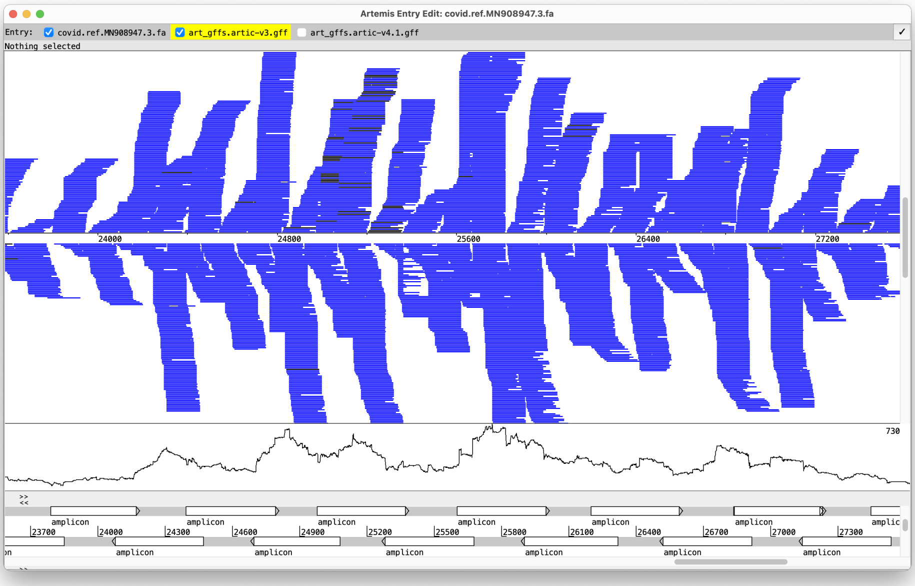

(b)

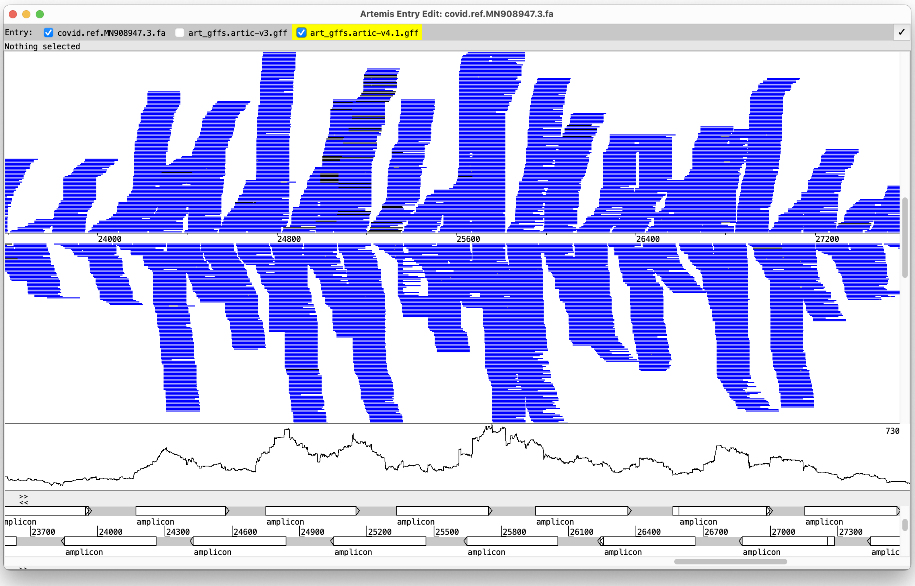

Figure 7: Artemis screenshots showing reads from Illumina run ERR8228569 mapped to the SARS-CoV-2 reference genome. (a) ARTIC amplicon scheme version 3 is annotated. (b) ARTIC amplicon scheme version 4 is annotated. See the legend of Supplementary Figure 7 for an explanation of the visualisation details. For this Illumina run, there is no clear match to either amplicon scheme.

4 Run time and memory

A summary of the run time and memory usage on the truth dataset is shown in figure 8. Values are taken from the output of the Unix command `/usr/bin/time`. Plots generated from the full results in Supplementary Table 5.

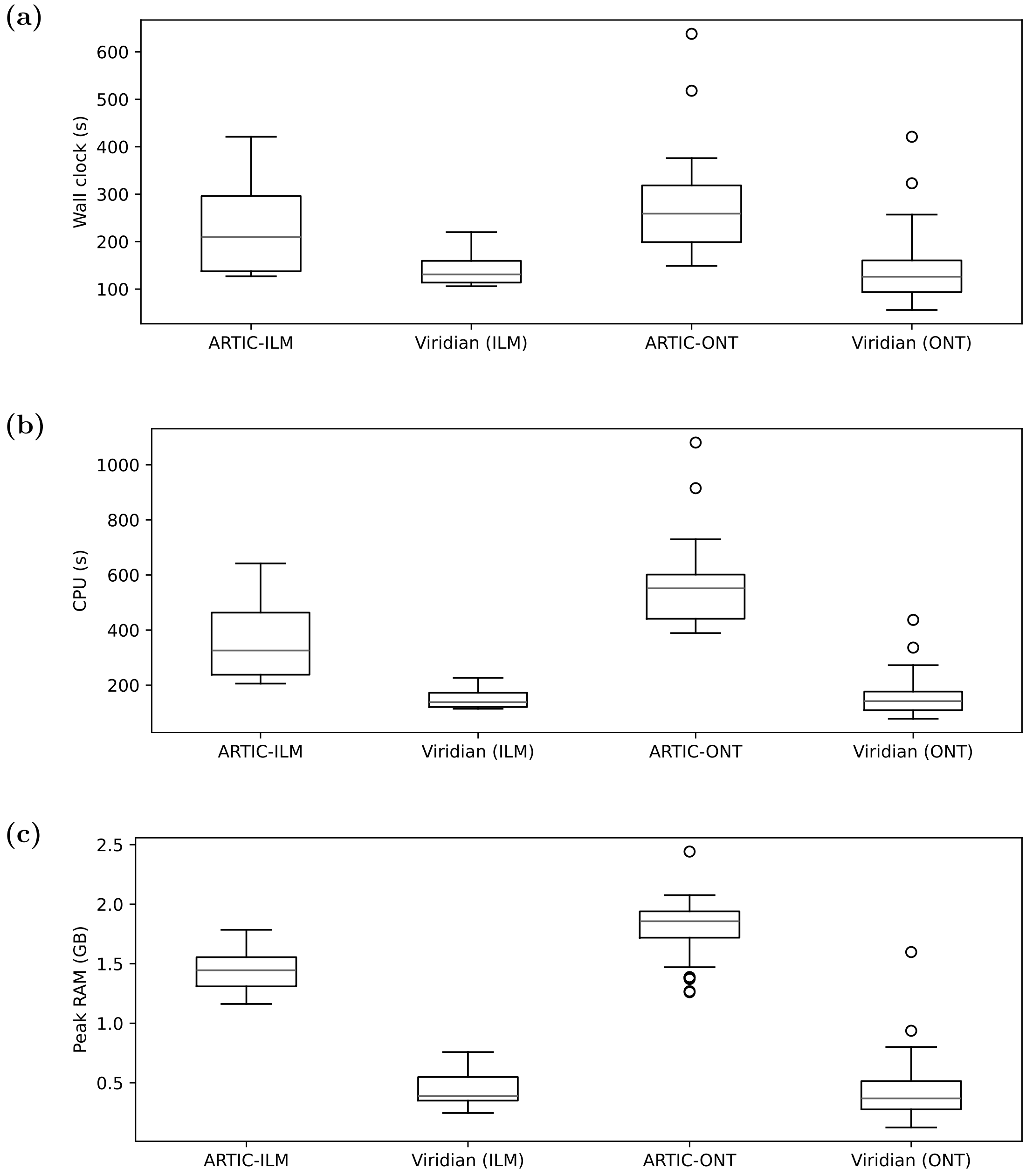

Figure 8: Comparison of a) wall clock time, b) total CPU time, and c) peak RAM usage on the truth dataset. Viridian results are split into Illumina and ONT, for comparison with the separate pipelines ARTIC-ILM and ARTIC-ONT.

5 Assembly and evaluation of the global data

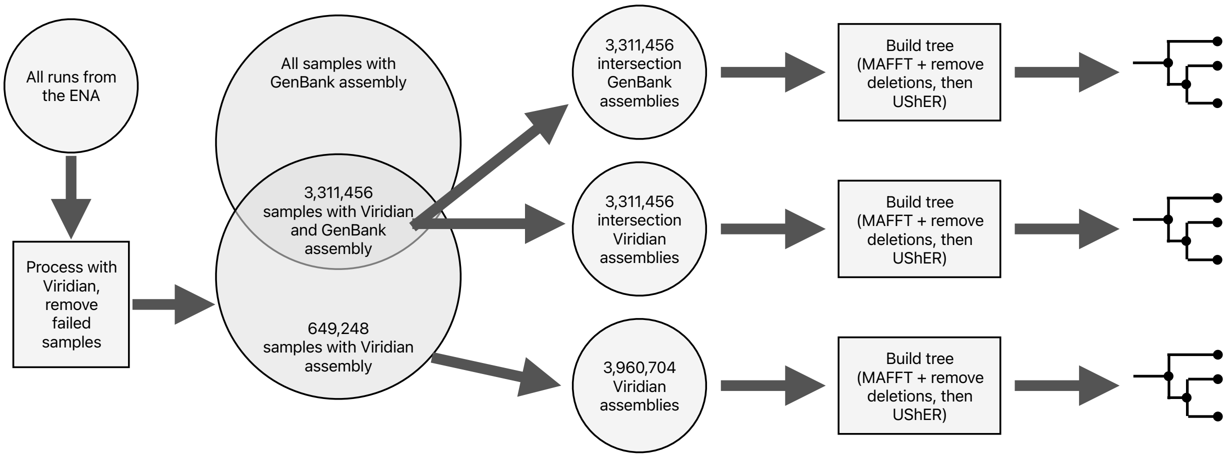

Figure 9: Sample processing and building of the global trees.

6 Indel calls

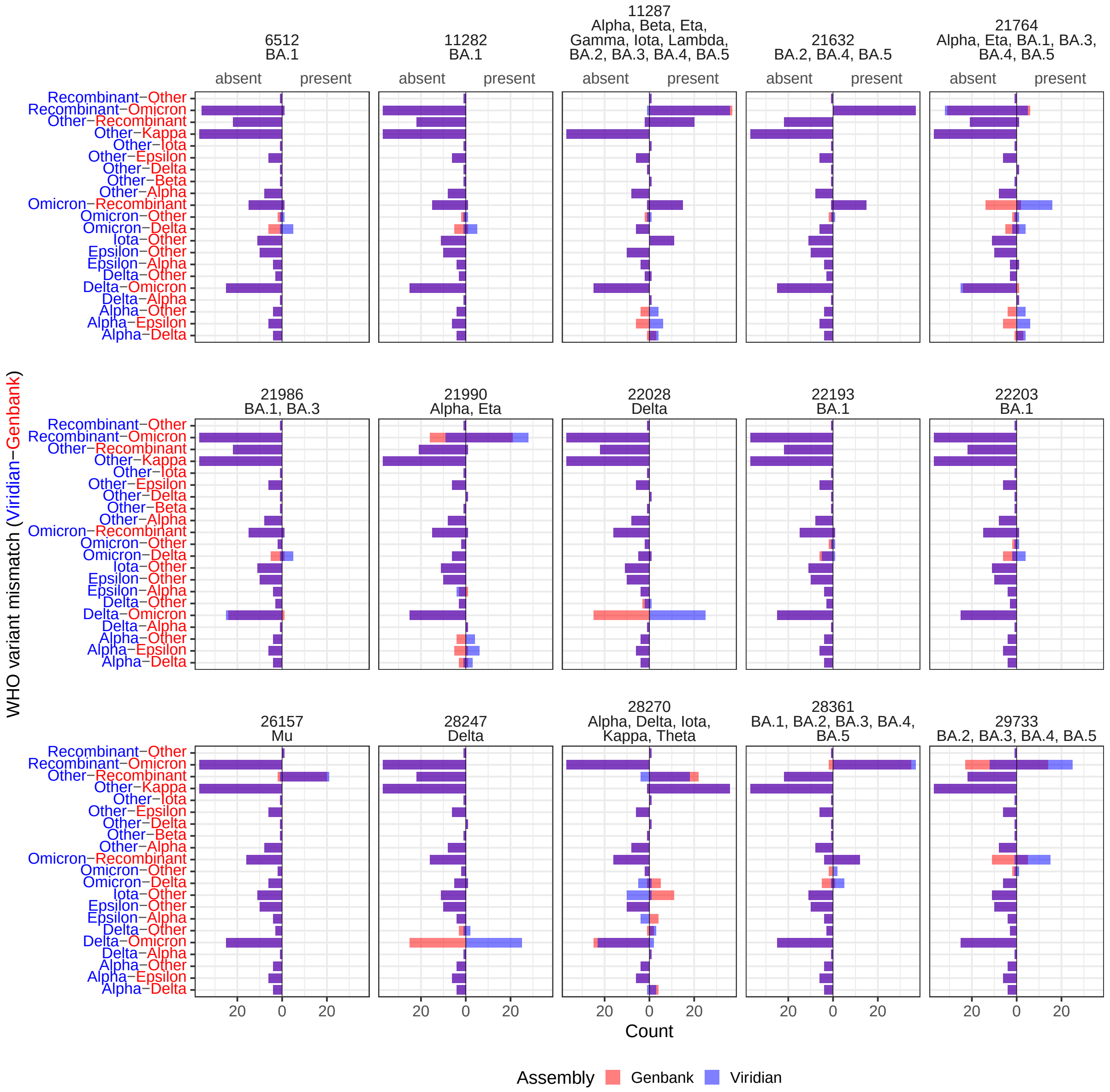

Figure 10: VOC-defining indels in samples where Viridian and GenBank disagree on Pango assignment. For (the few) genomes where the Pango WHO variant-of-concern assignment differed between Viridian and GenBank, for each defining indel within an official variant consensus, we compared the number of samples where the indel was not identified (left of black line) to that where it was (right of black line) using Viridian (blue) and Genbank (red). The purple bar overlap shows where the presence/absence is consistent between the two assemblies. The WHO variants in which the indel is consensus are listed under the site identifier. Overall the results are very consistent, with the biggest discrepancies being where Viridian identifies Delta-defining indels and the sample is called as Delta, whereas GenBank does not not call the indel, identifying the sample as Omicron.

7 Reversions

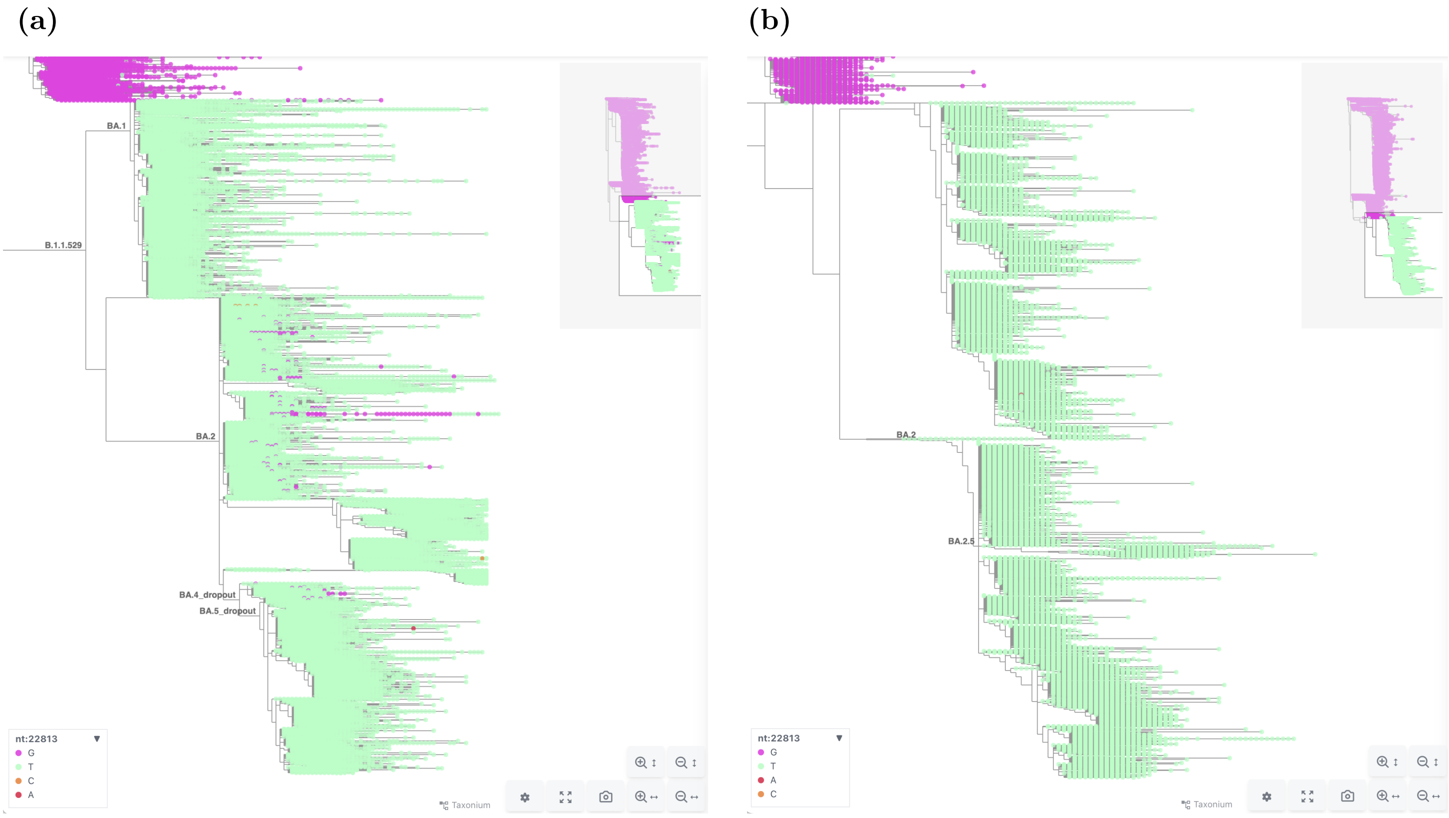

Figure 11: Taxonium screenshots of SARS-CoV-2 phylogenies, coloured by genotype at genome position 22813 (spike codon 417). a) The current UShER global phylogeny. b) The global Viridian phylogeny. Samples with the ancestral/reference genome allele are pink, and other genotypes (nearly all green) are shown in other colors.

8 Improved accuracy of lineage growth rate estimate

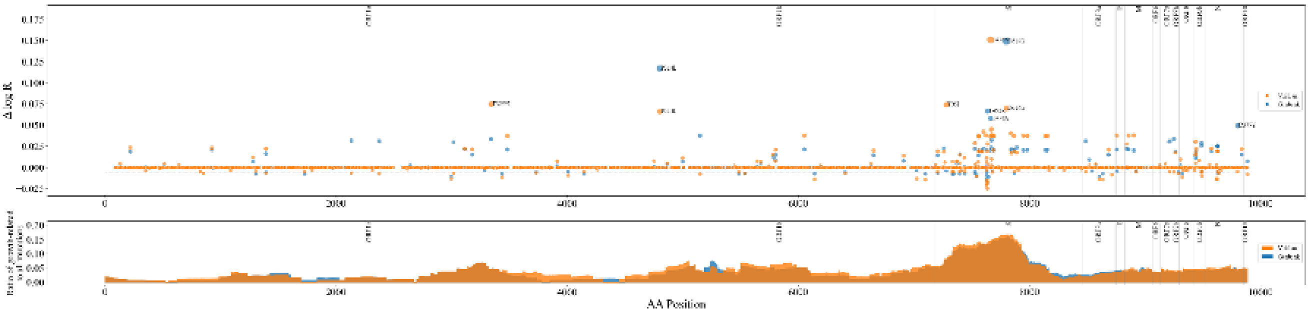

Figure 12: Manhattan plot showing mutation relative growth rate $\Delta \log R$ (y axis) by position (x axis) of mutations across the genome for each dataset, with reading frame annotated above. Relative growth rate $\Delta \log R$ is the contribution by a given mutation to the common log of the growth rate of a mutated strain divided by the growth rate of the ancestral strain. The 5 highest-growth mutations from each dataset are annotated. The standard deviation of mutation growth rates across both datasets is 0.006 – dotted lines at ± 0.006 are drawn to indicate growth-related mutations (mutations with $|\Delta \log R| > 0.006$). (b) The ratio of count of growth-related mutations to count of all mutations within a 600-amino-acid width window of x axis position is shown. Fisher's Exact Test is performed on the count of growth-related and non-growth-related mutations in each reading frame, with no statistically significant differences observed.

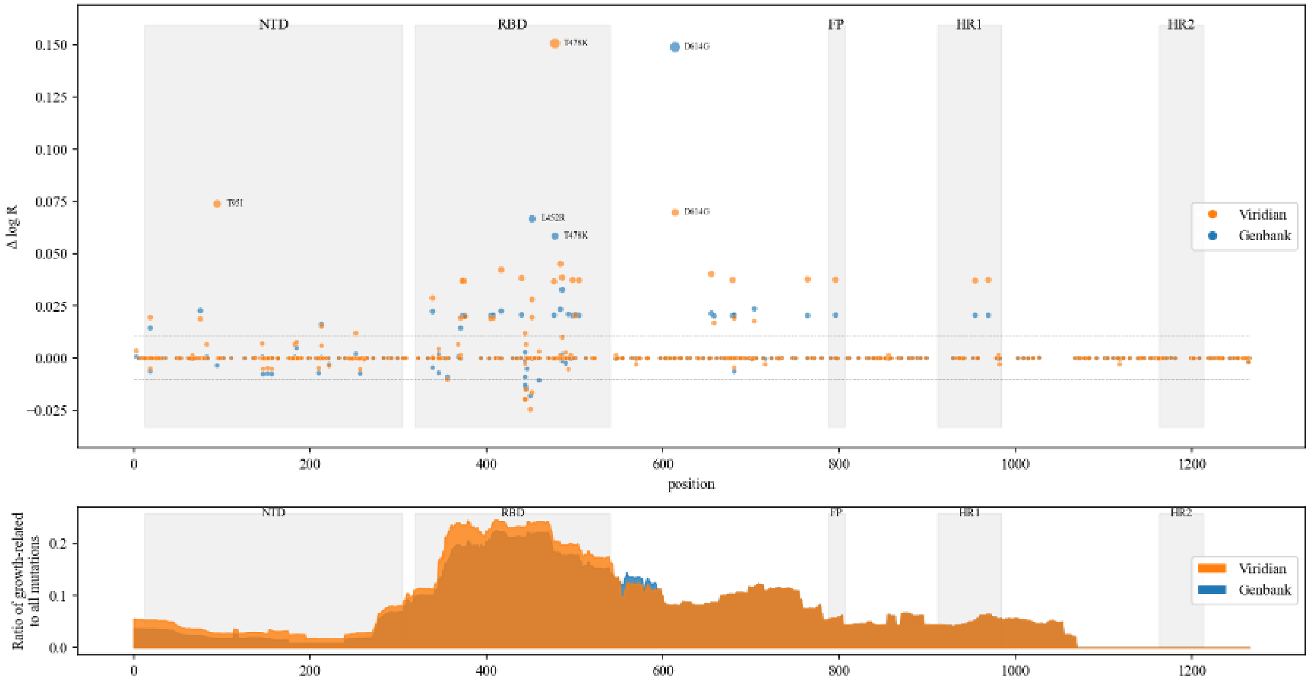

Figure 13: Mutation relative growth rate $\Delta \log R$ (y axis) by position (x axis) of mutations in the spike protein for each dataset, with 3 highest-growth mutations from each dataset annotated. Notably, when switching from GenBank to Viridian data, the growth rate of D614G approximately halves while the growth rate of T478K approximately doubles. (b) Ratio of count of growth-related mutations to count of all mutations within a 200-amino-acid width window of x axis position is shown. Each subregion (N-Terminal Domain (NTD), Receptor Binding Domain (RBD), Fusion Peptide (FP), Heptad Repeats 1 and 2 (HR1 and HR2)) is shaded and Fisher's Exact Test is performed for difference in proportions, yielding no statistically significant differences.

(a) **(b)**

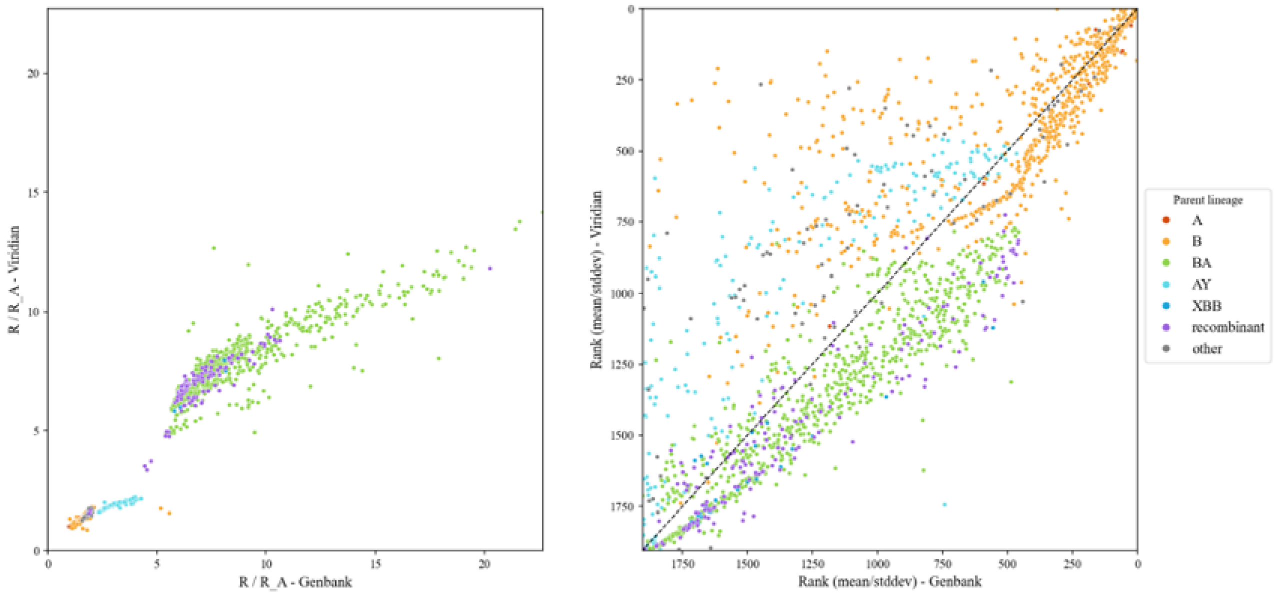

Figure 14: Note: legend labels denote parent lineage. (a) Relative growth rate of strain using Viridian data (y axis) versus GenBank data (x axis). Both datasets yield the result of growth rate clustering into two major clouds, mostly categorized by emergence of BA and recombinant lineages (and their sub-lineages). While we don't expect relative growth rate R/R_A to be exactly preserved across datasets (due to a different number of mutations, etc.), we do expect relative order to be consistent. (b) Rank of strain using Viridian data and GenBank data, where rank is determined by mean divided by standard deviation of growth rate posterior distribution. The dotted line $y = x$ is shown. Due to lower uncertainty estimates a posteriori using the Viridian data, there is a frequent shift of strains with poor rank using the GenBank data having better rank using the Viridian data, especially among B lineages and AY sub-lineages. This mean/stddev metric is common for feature selection, among other tasks. Since figure 4(a) shows that there is not much change in rank of mean R/R_A, we can attribute most of the changes in rank (mean/stddev) to changes in stddev. The points that lie above the y=x line are those for which uncertainty in the standard deviation of the R/R_A estimate likely decreased. This shows the power of Viridian in helping to decrease uncertainty values and prioritize different strains (notably AY and B) compared to GenBank.

9 Measuring uncertainty in the global tree

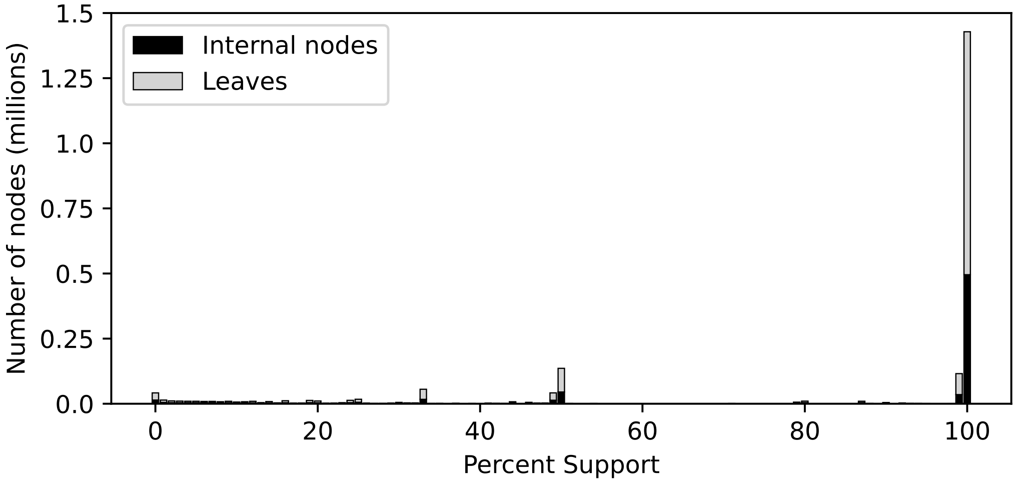

Figure 15: Histogram of support for each node in the global Viridian tree as measured by SPRTA. Bars are stacked, showing the support for internal nodes and for leaves in the tree.

(a)

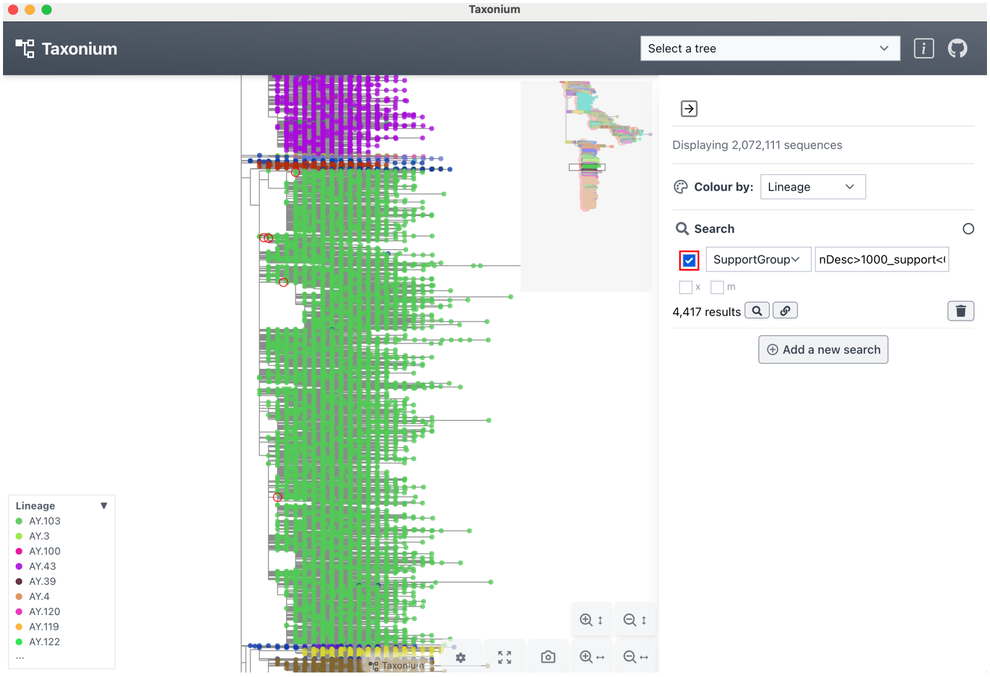

(b)

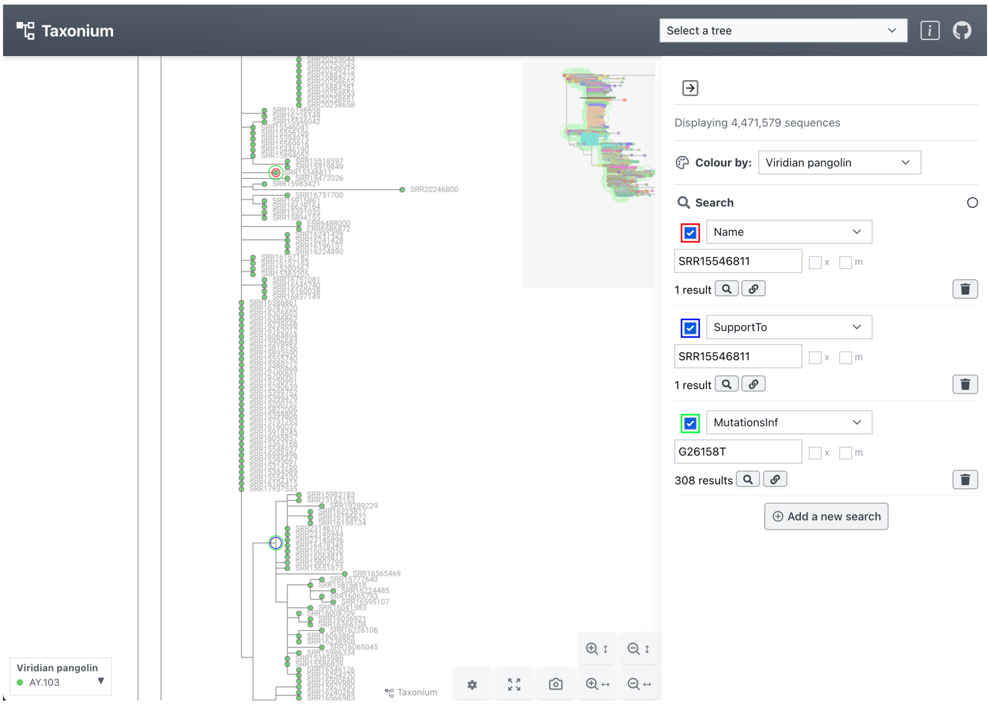

Figure 16: Screenshots of the global Viridian Maple/SPRTA tree viewed using Taxonium. Groups of nodes can be searched for uncertainty, and alternative placements shown for chosen nodes. a) The result of searching for nodes that are ancestral to 1,000-10,000 samples and that have SPRTA support less than 50%. These nodes are highlighted with red circles. b) SRR15546811, highlighted with the red circle, has 80% SPRTA support. Searching for the sample name in the feature "supportTo" shows the alternative placement of this sample, highlighted with the blue circle, which has the remaining 20% support. The uncertain placement is caused by the mutation G26158T occurring two times near each other in the tree. Positions of this mutation is indicated with cyan circles using the "MutationsInf" search (they are at the same nodes as the red and green nodes).

10 Impact on evolutionary and epidemiological analysis

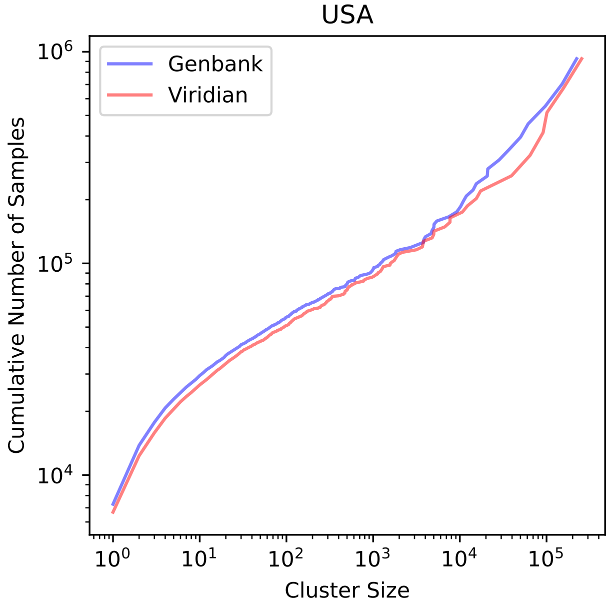

Figure 17: Cumulative Distribution of the number of samples in USA stratified by cluster size.

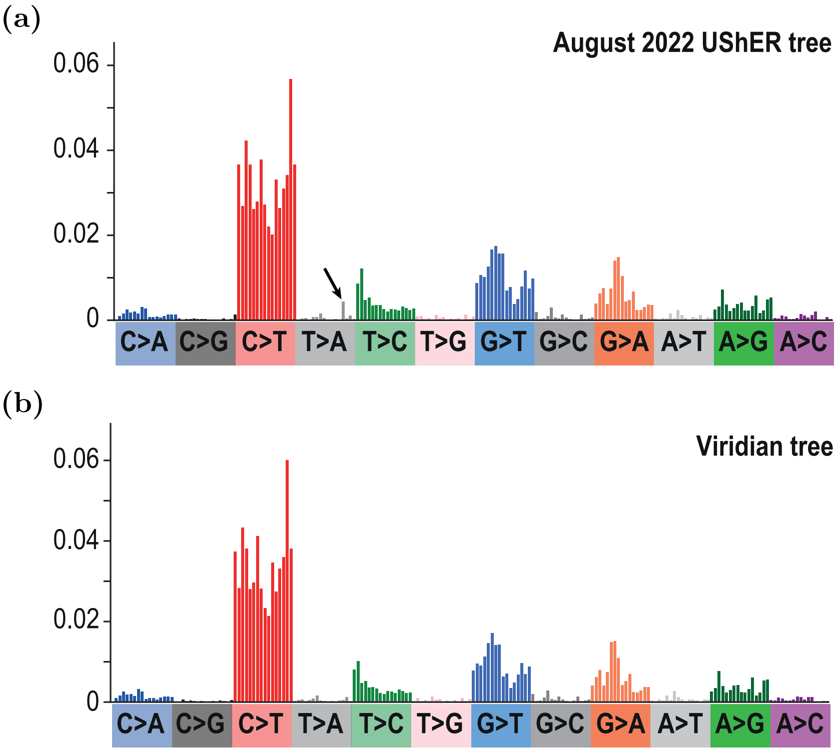

Figure 18: Comparison of Alpha variant mutational spectra calculated using (a) the August 2022 UShER tree [Ruis 2023] and (b) the Viridian tree. Colours show different mutation types (for example C mutating to T, labelled as C>T) and bars show individual surrounding contexts (for example an upstream A and a downstream A). Spectra are rescaled by the availability of the starting nucleotide triplet. The arrow shows a contextual mutation that is unexpectedly elevated in the August 2022 UShER tree; this elevation is not present in the Viridian tree.

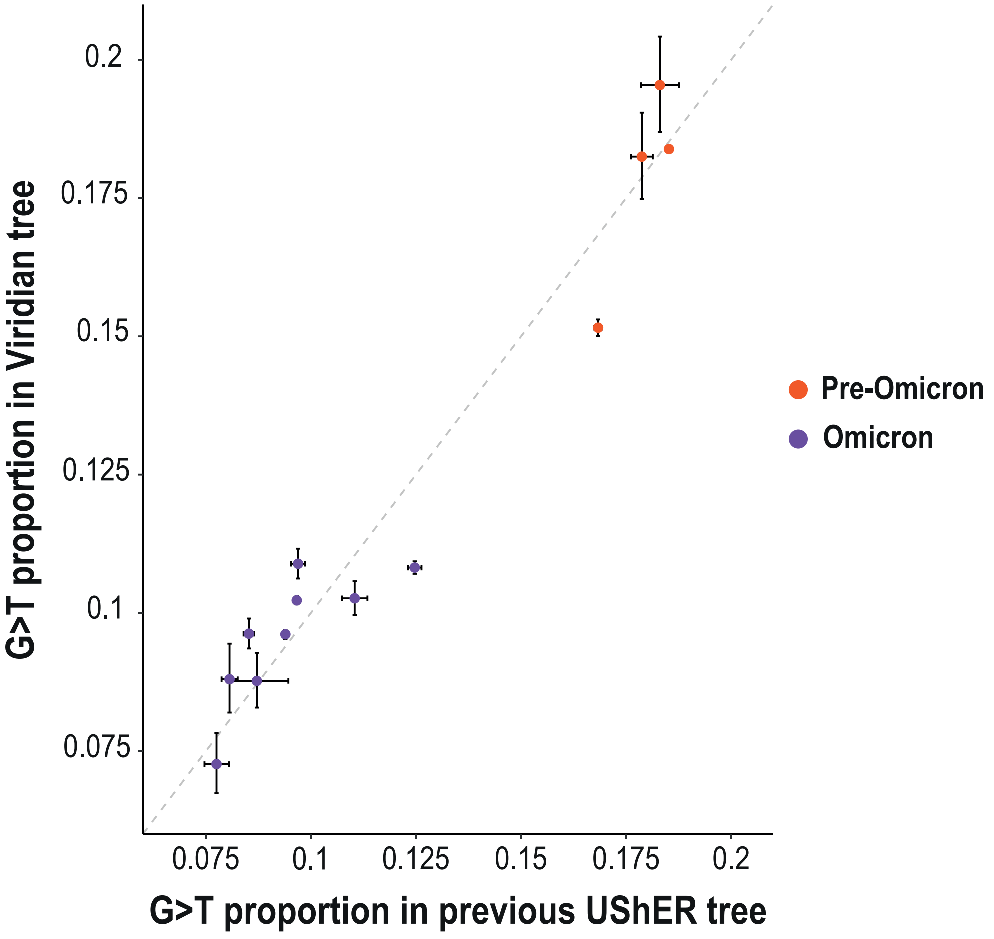

Figure 19: Comparison of the proportion of G>T mutations in Omicron and pre-Omicron SARS-CoV-2 lineages between previous UShER trees and the Viridian tree. Points show the proportion of G>T mutations and error bars show the Wilson score interval considering the calculated G>T proportion and number of sampled mutations. A previously observed reduction in G>T mutations in Omicron lineages [Ruis 2023] is still present in the Viridian tree. The date of the previous UShER tree depends on the lineage: August 2022 for Alpha, Beta, Gamma, Delta, BA.1, BA.2, BA.4 and BA.5; October 2023 for BA.2.12.1, BA.2.75, BQ.1, CH.1.1 and XBB.1.5.

11 mpox

We added the "Yale/Chen" amplicon scheme and reference genome MT903345 into Viridian and made a new release v1.5.1 with a new option `--species` that defaults to `sars-cov-2`. It can process mpox samples with `--species mpox`, which tells Viridian to use its built in mpox reference genome and the Yale/Chen amplicon scheme instead of SARS-CoV-2 data. Two fixes were required for mpox data. First, the mapping of each amplicon sequences – the output of racon – using minimap2 needed changing by running a second time with the option `-x asm20`, in addition to the existing run using `-x sr`. We found both runs were needed to get minimap2 to report all expected matches. Without this, some amplicons had no match and were rejected. Since viridian chooses the longest match on the forwards strand for each amplicon, any duplicate matches are handled. These changes to the use of minimap2 were applied regardless of the species option used.

The second change to Viridian was to use mafft to globally align the consensus sequence to the reference genome, because the existing method assumed no repeats in the genome, which is not true of the mpox genome. The default behaviour was updated so that if the reference genome is under 30kb then nothing changes, otherwise mafft is used for the global alignment.

We ran version 1.5.1 of Viridian on all samples from Chen et al that had sequencing runs in the ENA, which was 181 samples each with one Illumina paired sequencing run. Viridian rejected 5 samples because they failed the initial QC check requiring at least half of the genome to have at least 20X read depth. These were five of the six samples with the highest CT values (Supplementary Table 10 and Figure 20). The remaining samples all ran to completion and produced a consensus sequence. The pattern of dropped amplicons matched expectations, as shown in Supplementary Figure 21, considering the problematic amplicons highlighted by Chen et al (amplicon numbers 11, 26, 28, 56, 59, 60, 74, 75, 96, 118).

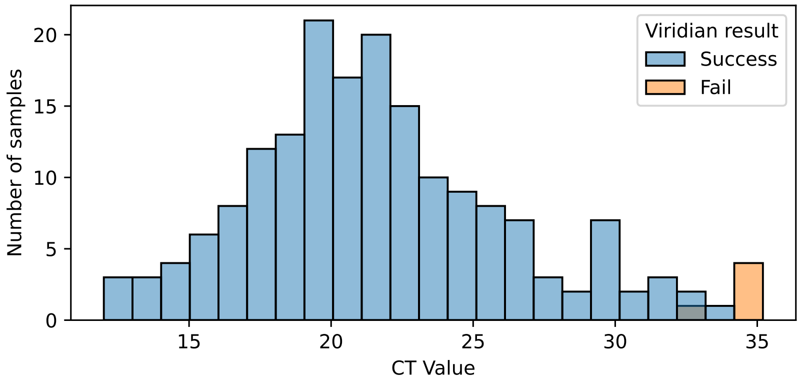

Figure 20: Distribution of CT values for the mpox samples, broken down into Viridian pass (blue) and fail (orange).

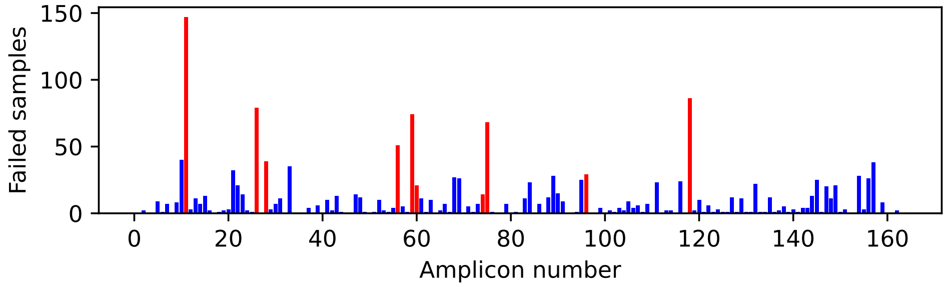

Figure 21: Number of times each amplicon was failed by Viridian in the mpox data. Known problematic samples (amplicon numbers 11, 26, 28, 56, 59, 60, 74, 75, 96, 118) highlighted by Chen et al are coloured red, and the rest are blue.

Since there is no truth for these samples, as a final sanity check we investigated the assembly lengths (Supplementary Figure 22). The amplicon scheme covers positions 357 to 196424 of the reference genome, totalling 196068bp. As a rough estimate of the expected length of each sample, `samtools depth -aa` was run on the BAM file made by Viridian. We took the start of the first 100bp window from with in amplicon scheme that had a minimum read depth of 20X as an estimate of where we would expect the assembly to start. Similarly, we took the end position to be the end of the first 100bp window from the end of the amplicon scheme with a minimum of 20X read depth. There is good correlation between this estimate and the length output by Viridian (Supplementary Figure 23), with a Spearman rank correlation of 0.7 and p-value of 1.67e$-$27.

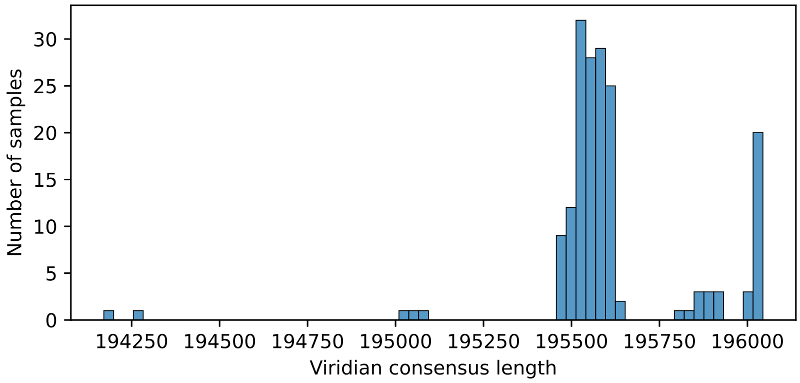

Figure 22: Histogram of length of consensus sequences output by Viridian on the mpox data.

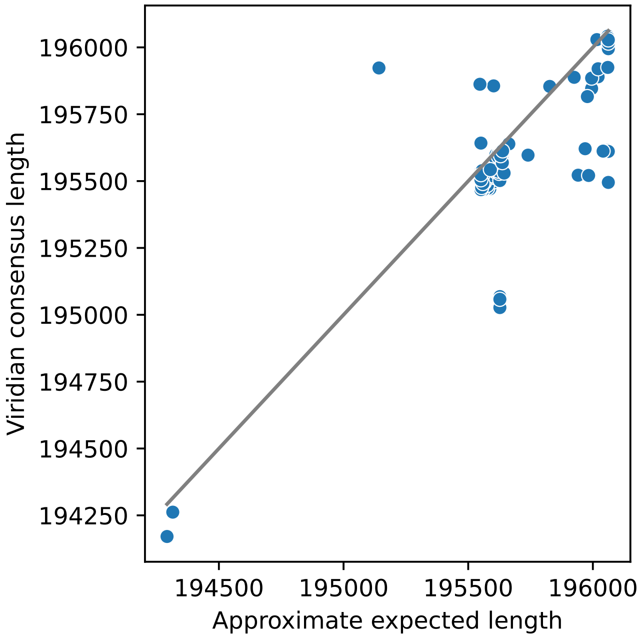

Figure 23: Scatter plot comparing the length of sequence output by Viridian and the expected length, for each sample in the mpox data. The diagonal line is $y = x$.

12 Geographical distribution of samples

The country for each sample was determined from the “Country” entry in the ENA metadata. The global Viridian tree produced in this study included all INSDC data up to 19th March 2024. The counts of samples for all countries are in Supplementary Table 11, the worldwide and Europe counts are shown in Supplementary figures 24 and 25.

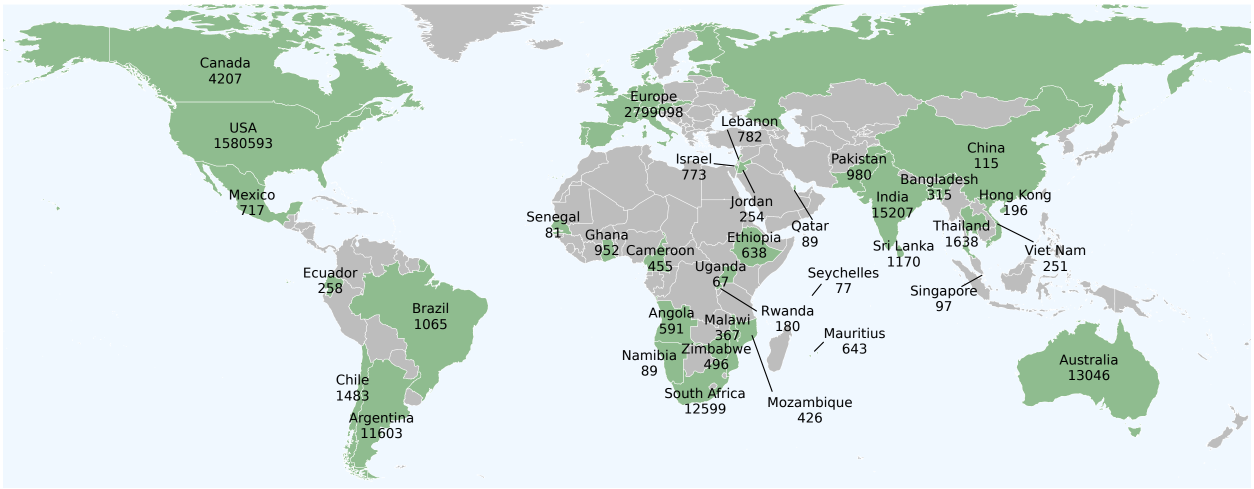

Figure 24: Worldwide geographical distribution of samples. Numbers show the total number of samples for each country, excluding QC failures, that are in the global Viridian tree. Only countries with at least 50 samples are labelled, and are coloured in green. See Supplementary Figure 25 for the per-country counts of Europe.

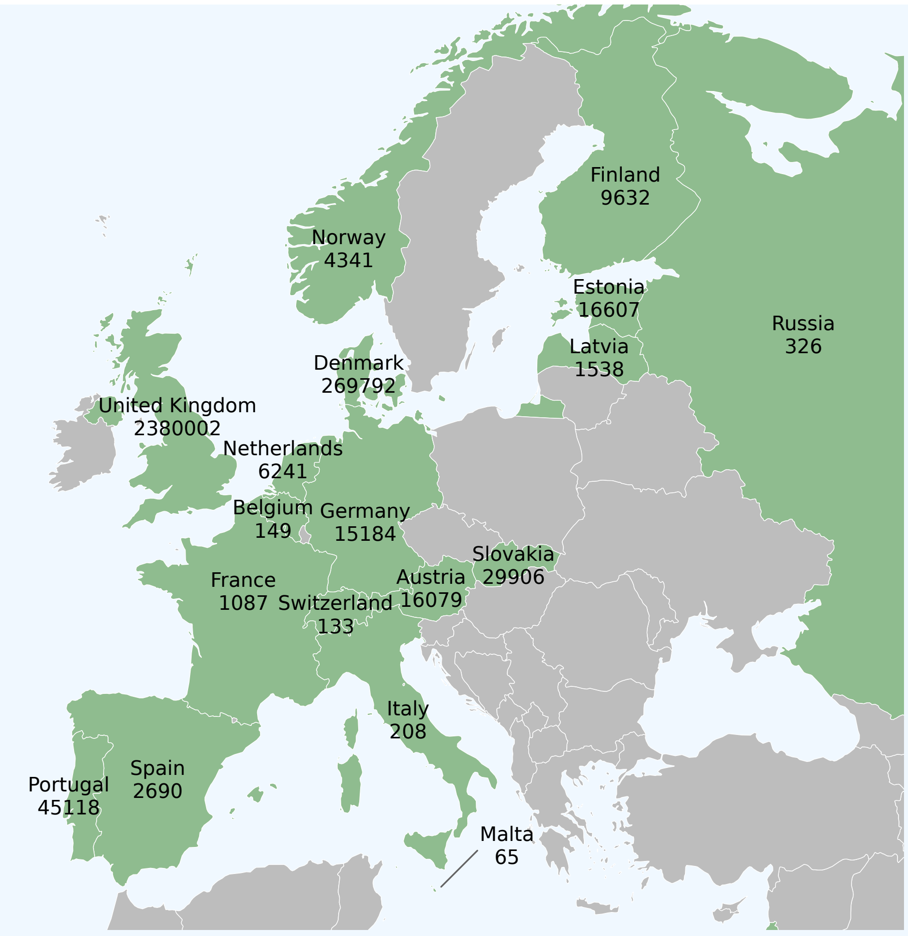

Figure 25: Geographical distribution of European samples. Numbers show the total number of samples for each country, excluding QC failures, that are in the global Viridian tree. Only countries with at least 50 samples are labelled, and are coloured in green. See Supplementary Figure 24 for worldwide counts.

13 Methods

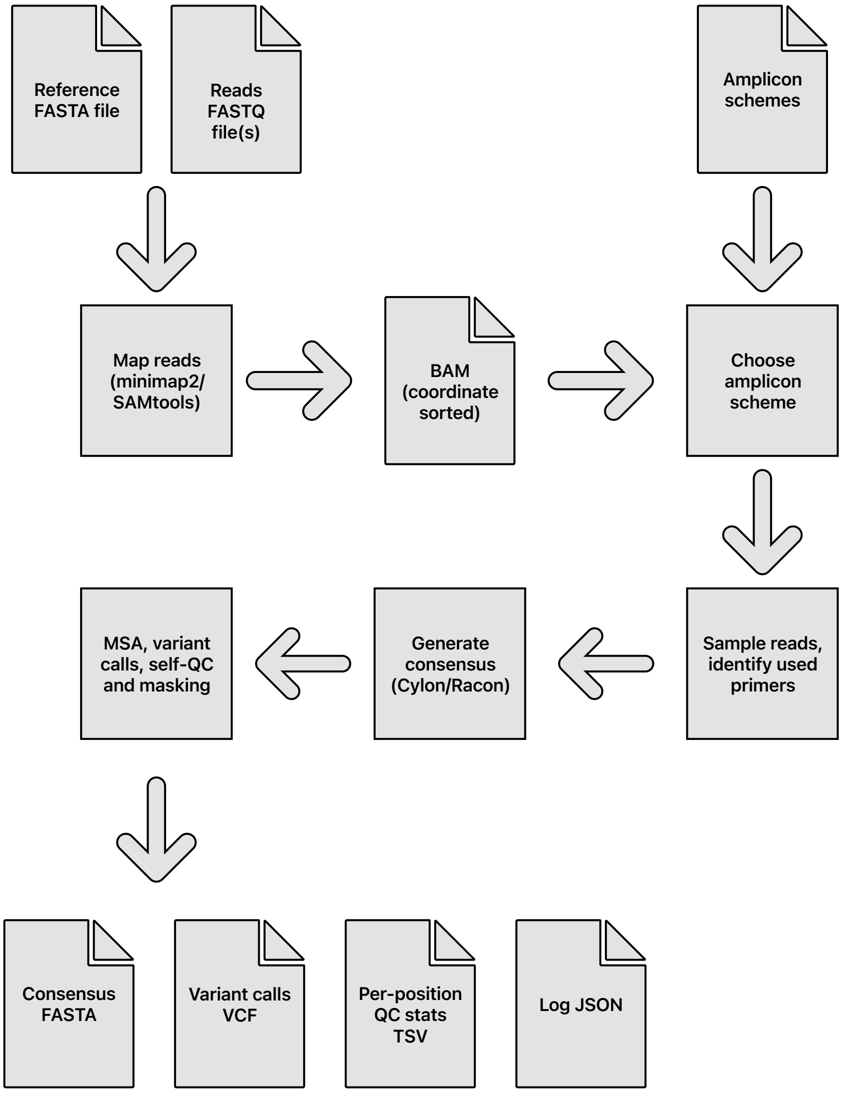

Figure 26: Overview of the Viridian pipeline, from input sequencing reads to output files.

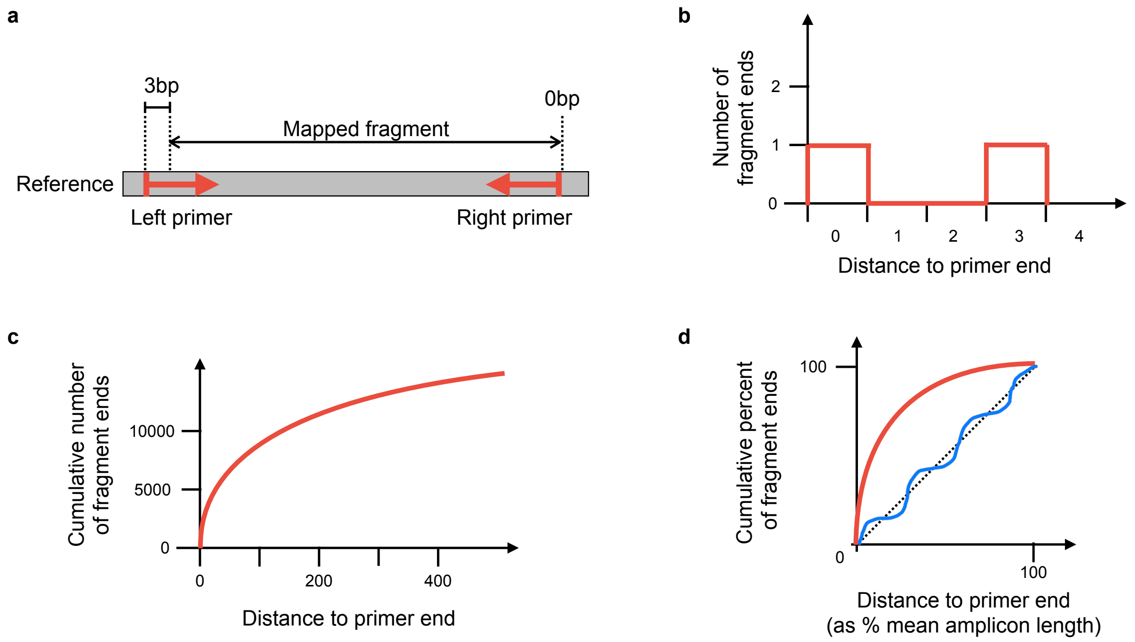

Figure 27: Method to score an amplicon scheme, using mapped fragments. **a)** Example of one mapped fragment, where its left end is 3bp from the start of the primer, and its right end is 0bp from the end of the right primer. **b)** The plot generated from the fragment in a). The right end of the fragment increments the counter for zero distance from a primer, and the left end of the fragment increments the counter for 3bp distance from a primer. The information from all fragments in the sample is added in this way, to make the distribution of distances from nearest primer ends. **c)** The cumulative plot from b) after adding all fragments. **d)** Plot c) is normalised by taking distance to primer end as a percentage of the mean amplicon length (x axis), and fragment counts as percent of total fragments (y axis). The red line indicates a typical curve where the reads match the scheme, whereas the blue line shows a scheme that does not match. The scheme's score is the sum of differences between the calculated line and the $y = x$ line (shown as a dashed line).

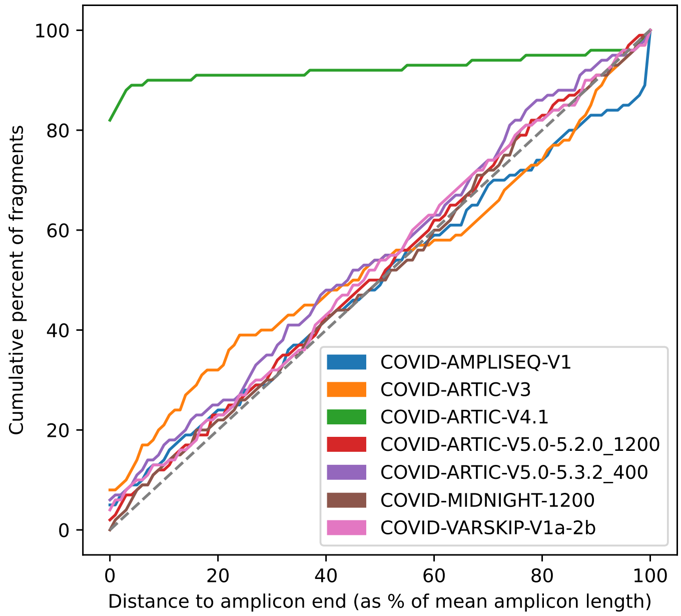

Figure 28: Example scheme identification score plot from Viridian. Made from run accession ERR8959196, which is Nanopore reads sequenced using ARTIC-V4.1 primers.

Amplicon scheme scores and cutoffs

Simulations

Viridian has a command line function to simulate error-free unpaired reads from all of its built-in amplicon schemes for a given read length, and then process those reads with the scheme identification/scoring code. Since these are unpaired reads, the read length is also the fragment length. For each scheme, Viridian simulates 50 unpaired reads from each amplicon. The genome position of the middle of the read is chosen randomly from a uniform distribution, and if the read would go past the start or end of the amplicon then it is trimmed to not exceed the range of the amplicon. Reads are forced to be at least 50bp long. It also simulates WGS reads by taking reads at 50X sampled randomly across the whole genome. Then the scheme identification is run for each set of reads, resulting in scheme scores for every set of reads and every scheme.

The simulations were run using fragmment/read lengths of 100, 120, 140, 160, 180, 200, 250, 300, 400, 500, 750, 1000, 1250, 1500, and 2000. The command used was for example with read length 100: `viridian sim_schemes --read_length 100 --outdir out.100`. A summary of the results is shown in Figure 29 and the raw data are in Supplementary Table 12.

By default, Viridian requires the best score to be more than 250, and the ratio of second best to best score to be less than 0.5. The results show that in simulations, WGS scores around 250 but the score ratio is in the range 0.6 to 0.8, meaning that all WGS simulations are rejected by Viridian's default filters.

The two amplicon schemes, Midnight-1200 and ARTIC 1200, with "long" amplicons - more than 1000bp - required relatively longer fragments to call the correct scheme and pass the default cutoffs. Midnight 1200 called the correct scheme with fragments of length at least 200bp, and also passed the filters with fragments at least 300bp. For ARTIC 1200, these numbers were 180bp and 250bp respectively. These schemes are usually used with nanopore reads, where each read is expected to span an entire amplicon, and such short fragment lengths are not expected. However, even if the sequencing involved fragmenting before sequencing, in most cases the sequenced fragments will be long enough. For simulations using the remaining amplicon schemes, with shorter amplicons, all schemes made the correct call and passed the filters with fragments of length at least 140bp.

Empirical truth data set

Plots comparing the best and second best scores on the empirical truth set are shown in Supplementary Figure 30, and the raw data are in Supplementary Table 13. The truth set includes SISPA sequencing, which results in reads randomly distributed across the genome and so should not agree with any of the amplicon schemes. This is analogous to the "WGS" simulated reads described above. All SISPA samples were rejected, and all other samples passed the default checks with the correct amplicon scheme called.

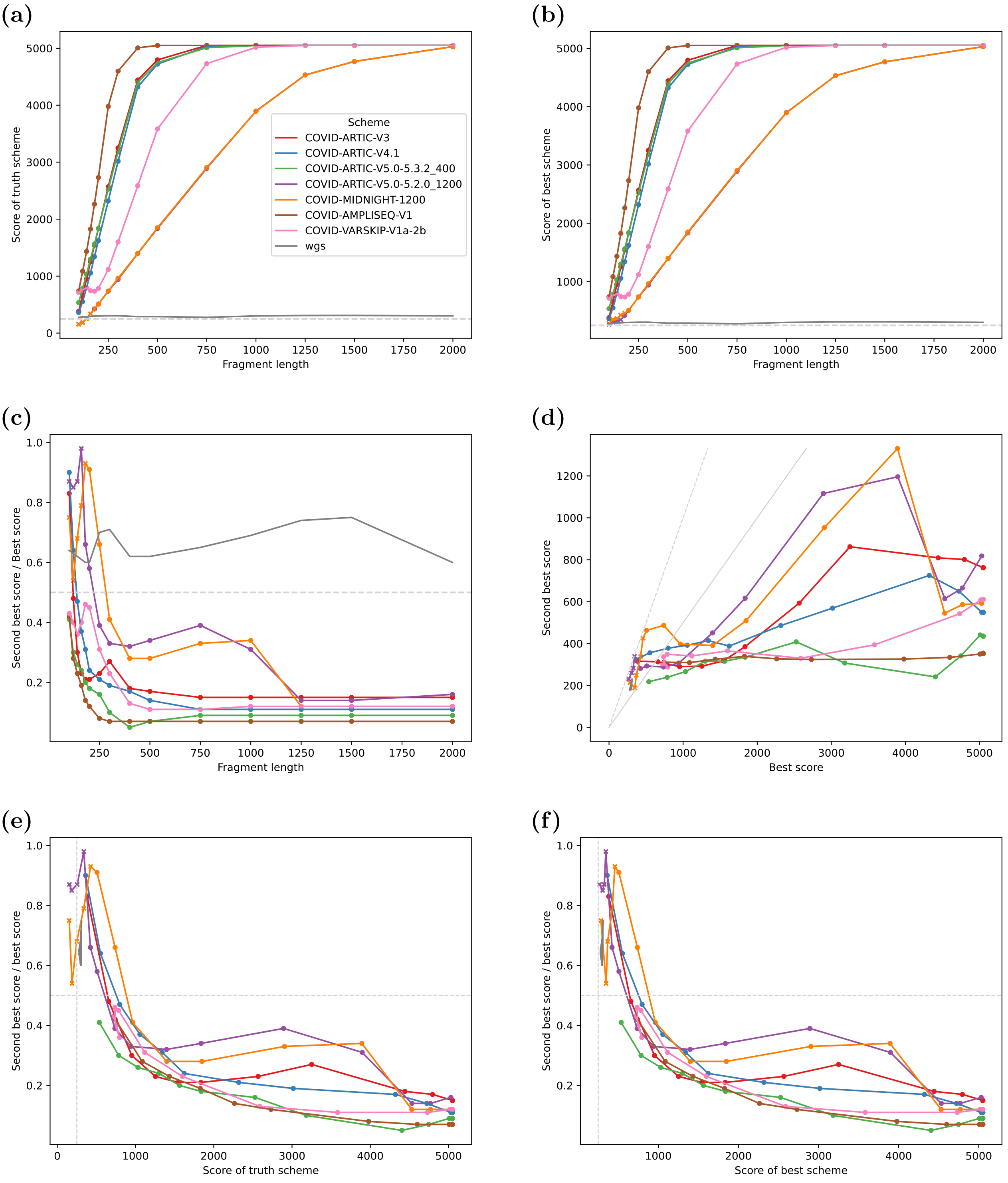

Figure 29: Viridian scheme identification results using simulated data. Crosses denote where the best score was for the wrong scheme, and circles for the correct scheme. Horizontal and vertical dashed lines show Viridian default cutoffs (pass is above horizontal and to the right of vertical lines). a) Score of the correct scheme as fragment length varies. b) Same as a), but the y axis shows the best score (i.e. the score of the highest scoring scheme as calculated by viridian, which may not be for the correct scheme). c) Second best / best score plotted against fragment length. d) Second best compared to best score. The dashed line shows $y = x$, and the solid line shows $y = 0.5x$, which is the cutoff for rejecting a sample if the second best score is more than half the best score. e) Second best / best score plotted against score of the correct scheme. f) Same as e), but the x axis shows the best score instead of the score of the correct scheme.

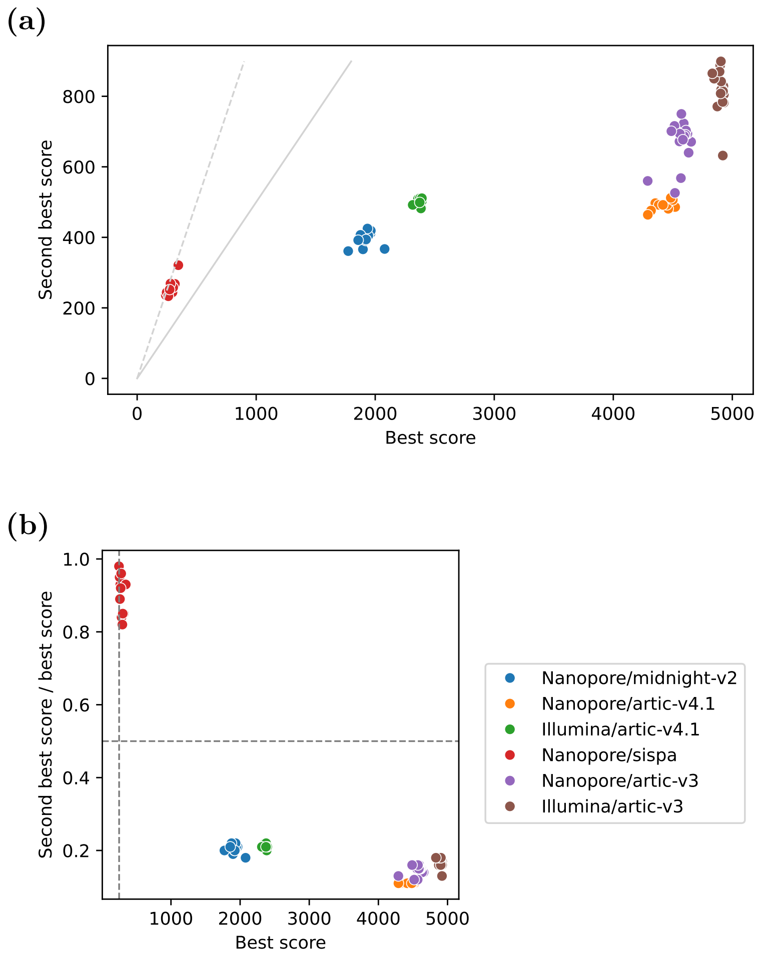

Figure 30: Scores of Viridian's scheme identification on the empirical truth dataset. a) Scatter plot of the second best score against the best score. The dashed line shows $y = x$, and the solid line shows $y = 0.5x$, which is the cutoff for rejecting a sample if the second best score is more than half the best score. b) Scatter plot of the ratio of second best / best score, against best score. The dashed lines show the default cutoffs: if the best score is less than 250 or the score ratio is more than 0.5 then the sample is rejected.

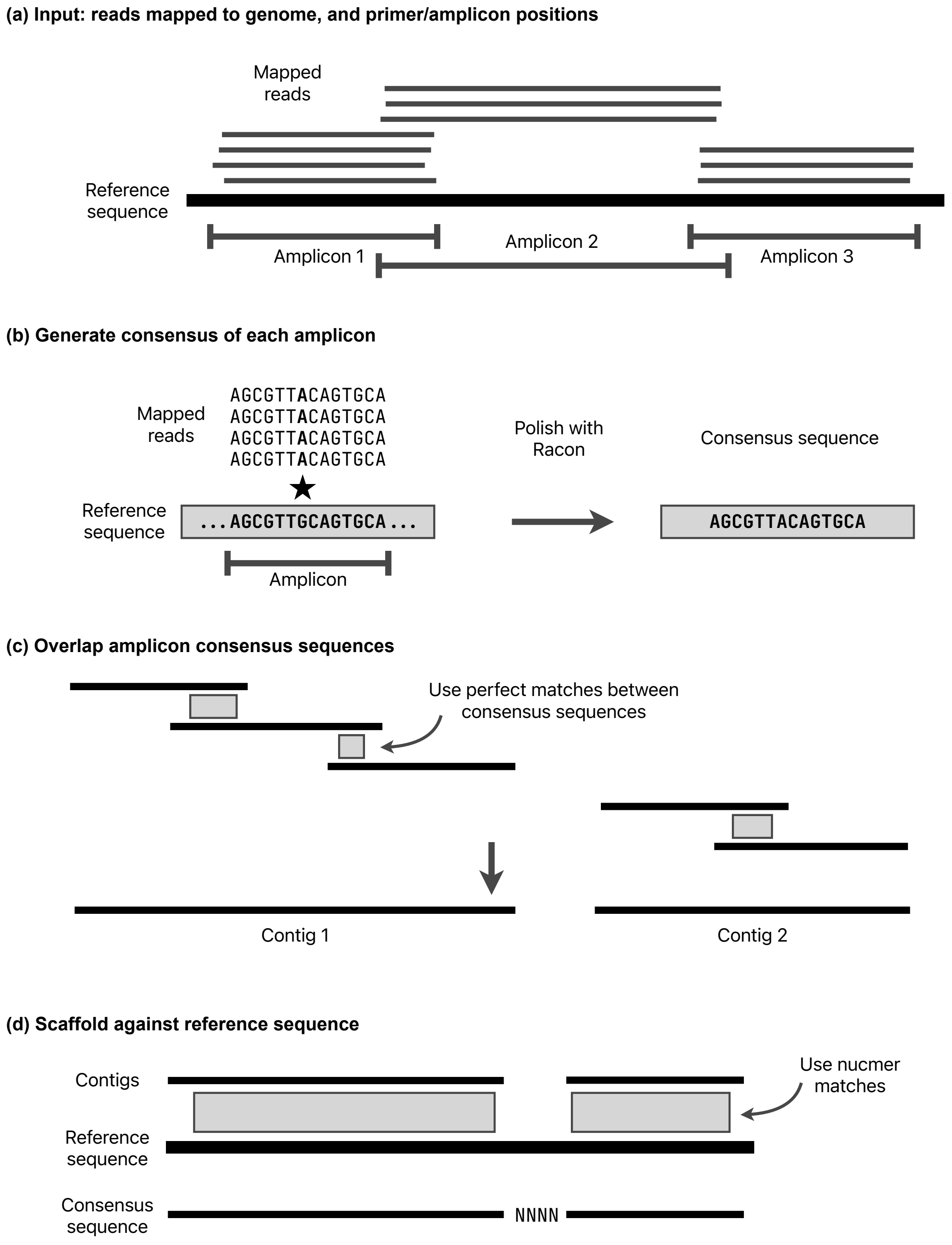

Figure 31: Consensus sequence construction methods. See main text for details. a) The starting point is primer and amplicon positions, and reads mapped to the consensus sequence. b) The consensus sequence of each amplicon is generated independently, using Racon. c) The amplicon sequences are overlapped using perfect matches (if they exist), making contigs. d) The contigs are scaffolded against the reference genome, adding gaps where needed.

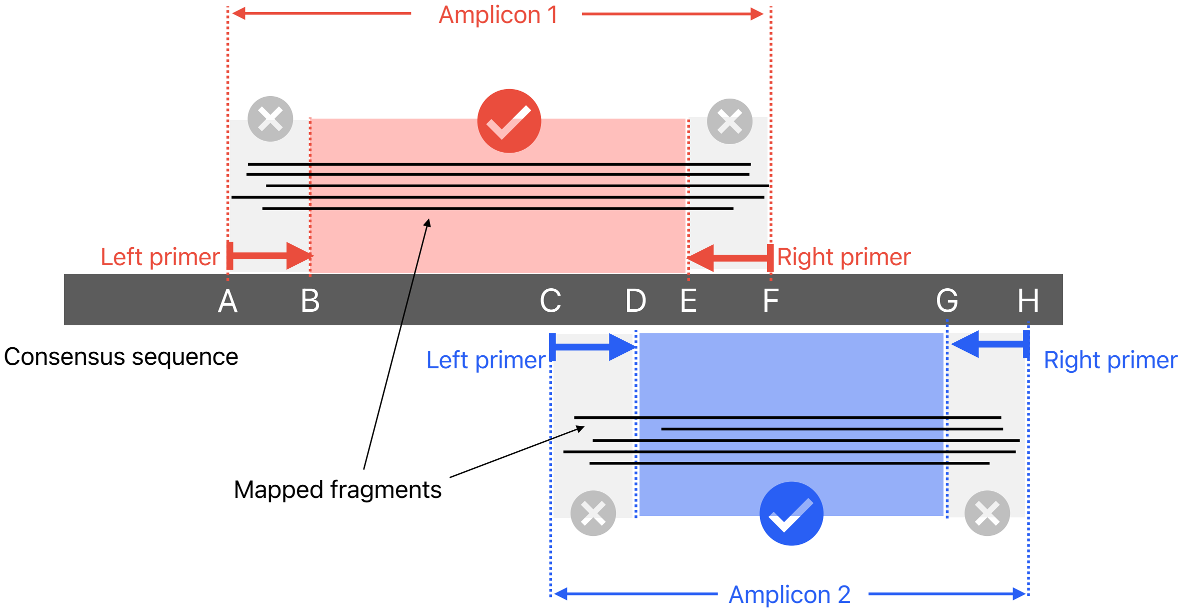

Figure 32: Consensus sequence pileup/masking methods. Two amplicons are shown with fragments (either illumina read pairs, or unpaired nanopore reads) mapped to the consensus. The fragments from amplicon 1 contribute to pileup at B-E, and do not count towards the primer regions A-B or E-F. Similarly, the fragments from amplicon 2 contribute to coverage at D-G (but not to C-D or G-H).

14 IMSSC2 Laboratory Network Consortium members

Names	Institution
Barbara Biere, Ralf Dürrwald, Christin Mache, Djin-Ye Oh, Jessica Schulze, Marianne Wedde, Thorsten Wolff	Unit "Influenza and other Respiratory Viruses", RKI, Berlin, Germany
Stephan Fuchs, Torsten Semmler, Sofia Paraskevopoulou	Unit "Genome Competence Centre", RKI, Berlin, Germany
Romy Kerber, Stefan Kröger, Walter Haas	Unit "Respiratory Infections", RKI, Berlin, Germany
Konrad Bode	MVZ Labor Dr Limbach, Heidelberg, Germany
Victor Corman	Institute of Virology, Charité-University Medicine, Berlin, Germany
Michael Erren	MVZ Laborzentrum Weser-Ems
Patrick Finzer	MVZ Düsseldorf-Centrum, Düsseldorf, Germany
Roger Grosser	Labor Dr Wisplinghoff, Köln
Manuel Haffner	MVZ Labor Dr Kirkamm, Mainz, Germany
Beate Hermann	MVZ Dianovis, Greiz, Germany
Christina Kiel	MVZ Labor Dessau, Dessau-Roßlau, Germany
Andi Krumbholz, Thomas Lorentz	Labor Dr Krause, Kiel, Germany
Kristian Meinck	IMD-Laborverbund, Greifswald, Germany
Andreas Nitsche	Unit "Highly pathogenic viruses", RKI, Berlin, Germany
Markus Petzold	Institut für Medizinische Mikrobiologie und Hygiene, Institut für Virologie, TU Dresden, Germany
Thomas Schwanz	Institut für Medizinische Mikrobiologie und Hygiene, Universitätsmedizin Mainz, Germany
Florian Szabados	Laborarztpraxis Osnabrück, Georgsmarienhütte, Germany
Friedemann Tewald	Labor Enders, Stuttgart, Germany
Carsten Tiemann	Labor Krone, Bad Salzuflen, Germany
