# Supplementary material for: Addressing pandemic-wide systematic errors in the SARS-CoV-2 phylogeny
Source: Nat Methods. 2026 Feb 9;23(3):653–62. doi: 10.1038/s41592-025-02947-1 (PMC12982125; doi:10.1038/s41592-025-02947-1)
Supplement: Supplementary file 2 — Reporting Summary [file 41592_2025_2947_MOESM2_ESM.pdf]

nature portfolio

Corresponding author(s): Zamin Iqbal

Last updated by author(s): 10/21/2025

Reporting Summary

Nature Portfolio wishes to improve the reproducibility of the work that we publish. This form provides structure for consistency and transparency in reporting. For further information on Nature Portfolio policies, see our Editorial Policies and the Editorial Policy Checklist.

Statistics

For all statistical analyses, confirm that the following items are present in the figure legend, table legend, main text, or Methods section.

n/a	Confirmed	
☐	☒	The exact sample size (*n*) for each experimental group/condition, given as a discrete number and unit of measurement
☐	☒	A statement on whether measurements were taken from distinct samples or whether the same sample was measured repeatedly
☐	☒	The statistical test(s) used AND whether they are one- or two-sided<br>*Only common tests should be described solely by name; describe more complex techniques in the Methods section.*
☒	☐	A description of all covariates tested
☒	☐	A description of any assumptions or corrections, such as tests of normality and adjustment for multiple comparisons
☐	☒	A full description of the statistical parameters including central tendency (e.g. means) or other basic estimates (e.g. regression coefficient) AND variation (e.g. standard deviation) or associated estimates of uncertainty (e.g. confidence intervals)
☐	☒	For null hypothesis testing, the test statistic (e.g. *F*, *t*, *r*) with confidence intervals, effect sizes, degrees of freedom and *P* value noted<br>*Give P values as exact values whenever suitable.*
☒	☐	For Bayesian analysis, information on the choice of priors and Markov chain Monte Carlo settings
☒	☐	For hierarchical and complex designs, identification of the appropriate level for tests and full reporting of outcomes
☒	☐	Estimates of effect sizes (e.g. Cohen's *d*, Pearson's *r*), indicating how they were calculated

Our web collection on statistics for biologists contains articles on many of the points above.

Software and code

Policy information about availability of computer code

Data collection: We downloaded all tiled amplicon SARS-CoV-2 sequence data from the ENA/SRA as of June 2024.

Data analysis: Package versions used for the simulations were: Snakemake v7.8.5, PhastSim v0.0.4, ART v2016.06.05, Badread git commit c2bdcbe, ARTIC Illumina workflow git commit 8af5152 from https://github.com/connor-lab/ncov2019-artic-nf, Epi2me wf-artic git commit 218aa1d from https://github.com/epi2me-labs/wf-artic, CTE git commit 9cd94b8 from https://github.com/iqbal-lab-org/covid-truth-eval, Nextflow v21.04.3, bwa git commit c77ace7, htslib v1.14, samtools v1.14, BEDTools v2.30.0, joblib v1.1.0 from https://github.com/joblib/joblib, numpy v1.22.1, pandas v1.4.0, pysam v0.18.0 at https://github.com/pysam-developers/pysam tq,dm v4.62.3 from https://github.com/tqdm/tqdm. Version 4.3 of Pangolin, and version 1.21 of pangolin-data were used for the intersection dataset. Version 1.29 of pangolin-data was used on the final Viridian global tree. MAPLE version 0.7.2 was used to measure uncertainty in the global tree. Viridian v1.0.0 or v1.1.0 was used to process all runs. The only difference between these versions is v1.1.0 added support for unpaired Illumina reads. The versions of tools used by Viridian were: Cylon git commit 57d559a, minimap2 git commit b0b199f, MUMmer v4.0.0rc1, NGMerge git commit 224fc6a, Racon git commit a2cfcac, Varifier git commit 8bc8726. Ushonium git commit b024320 was used, with dependencies MAFFT v7.520, UShER git commit 2df81ee, and taxoniumtools v2.0.111. We also ran version 3.10 of pyR0 from https: //github.com/broadinstitute/pyro-cov?tab=readme-ov-file.

For manuscripts utilizing custom algorithms or software that are central to the research but not yet described in published literature, software must be made available to editors and reviewers. We strongly encourage code deposition in a community repository (e.g. GitHub). See the Nature Portfolio guidelines for submitting code & software for further information.

Data

Policy information about availability of data

All manuscripts must include a data availability statement. This statement should provide the following information, where applicable:

- Accession codes, unique identifiers, or web links for publicly available datasets
- A description of any restrictions on data availability
- For clinical datasets or third party data, please ensure that the statement adheres to our policy

Supplementary text and figures S1-9 are in the supplementary PDF file.

The global Viridian tree is hosted at https://viridian.taxonium.org.

All other additional files are available from Figshare:
• Supplementary table S1[60], https://doi.org/10.6084/m9.figshare.27195261 - this is a TSV file containing metadata of all 5,959,032 sequencing runs considered in this study
• Supplementary tables S2-15 in one xlsx file[61], https://doi.org/10.6084/m9.figshare.28987784, details below:

S2 - Summary of counts of amplicon schemes in INSDC metadata and the scheme called by Viridian
S3 - Accuracy of Viridian, ARTIC-ILM and ARTIC-ONT on simulated data
S4 - Accuracy of Viridian, ARTIC-ILM and ARTIC-ONT on Illumina truth data set
S5 - Accuracy of Viridian, ARTIC-ILM and ARTIC-ONT on Nanopore truth data set
S6 - Run times and RAM usage on the truth data set
S7 - Metadata for the African data set
S8 - Counts of sites with errors in the African data set
S9 - Confidence of nodes in the global viridian tree
S10 - Numbers of inferred viral introductions
S11 - mpox data
S12 - Country counts in the Viridian global tree, and number of new samples since the tree was built
S13 - Viridian amplicon scheme scores using simulated data
S14 - Viridian amplicon scheme scores on the truth data set
S15 - Positions masked when building the global Viridian tree

• Supplementary HTML file, https://doi.org/10.6084/m9.figshare.25713198-comparison of Viridian and GenBank assemblies
• All Viridian consensus sequences that are in the global tree, split over two tar archive files (https://doi.org/10.6084/m9.figshare.25713225, https://doi.org/10.6084/m9.figshare.27194637), which contain the sequences split over multiple xzipped FASTA files. These are the same batched FASTA files used when building the trees.
• The Viridian global tree of 4,471,579 sequences, in JSONL and .pb format[65], https://doi.org/10.6084/m9.figshare.27194547
• The GenBank and Viridian intersection trees in JSONL and .pb format[66], https://doi.org/10.6084/m9.figshare.25713285
• All other Viridian consensus sequences that are not in the global tree, split over two xzipped FASTA files - https://doi.org/10.6084/m9.figshare.25713342, https://doi.org/10.6084/m9.figshare.27194652.
• The output TSV file from Maple/SPRTA run on the global Viridian tree[69], https://doi.org/10.6084/m9.figshare.28985573.v1
• The Viridian global tree with Maple/SPRTA data added, in JSONL format[70], https://doi.org/10.6084/m9.figshare.29097608

Human research participants

Policy information about studies involving human research participants and Sex and Gender in Research.

Reporting on sex and gender	N/A
Population characteristics	N/A
Recruitment	N/A
Ethics oversight	N/A

Note that full information on the approval of the study protocol must also be provided in the manuscript.

Field-specific reporting

Please select the one below that is the best fit for your research. If you are not sure, read the appropriate sections before making your selection.

☒ Life sciences ☐ Behavioural & social sciences ☐ Ecological, evolutionary & environmental sciences

For a reference copy of the document with all sections, see nature.com/documents/nr-reporting-summary-flat.pdf

Life sciences study design

All studies must disclose on these points even when the disclosure is negative.

Sample size	We analyse all public tiled-amplicon genome data for SARS-CoV-2, about 6 million genomes.
Data exclusions	None (except for our criteria that we only analyse tiled amplicon data)
Replication	We measure the error rates of different software processes (amplicon assembly by Viridian and state-of-the-art alternatives), first on simulated data (n=8000), then a small manually curated truth set of 67 genomes, then a larger independent dataset (Early Omicron data from Africa, n=12,287), and then via various indirect methods (number of reversions in the tree, comparison of amplicon scheme identification with metadata) on further independent datasets (all other sequenced SARS-CoV-2). In doing so we take care to consider key covariates (sequencing technology, primer scheme version, and also Pango/lineage). This is in a sense replication of those error estimates. But beyond that, this is not a paper making claims that A causes B, or is predictive of B, so there is no replication in the normal sense of for example GWAS.
Randomization	N/A. There is no randomization because we are not selecting from a population, or assigning to different groups, or comparing groups, or doing experiments.
Blinding	There was no blinding because there was no group allocation. This is not a study where we compare groups.

Reporting for specific materials, systems and methods

We require information from authors about some types of materials, experimental systems and methods used in many studies. Here, indicate whether each material, system or method listed is relevant to your study. If you are not sure if a list item applies to your research, read the appropriate section before selecting a response.

Materials & experimental systems

n/a	Involved in the study	
☒	☐	Antibodies
☒	☐	Eukaryotic cell lines
☒	☐	Palaeontology and archaeology
☒	☐	Animals and other organisms
☒	☐	Clinical data
☒	☐	Dual use research of concern

Methods

n/a	Involved in the study	
☒	☐	ChIP-seq
☒	☐	Flow cytometry
☒	☐	MRI-based neuroimaging

March 2021
