# Supplementary material for: Addressing pandemic-wide systematic errors in the SARS-CoV-2 phylogeny
Source: Nat Methods. 2026 Feb 9;23(3):653–62. doi: 10.1038/s41592-025-02947-1 (PMC12982125; doi:10.1038/s41592-025-02947-1)
Supplement: Supplementary file 4 — Comparison of Viridian and GenBank assemblies [file 41592_2025_2947_MOESM4_ESM.html]

Comparison of Viridian and Genbank assemblies


# Comparison of Viridian and Genbank assemblies

### Bethany Dearlove, Lukas Endler and Andreas Bergthaler

#### Institute for Hygiene and Applied Immunology, Medical University of Vienna Contact: bethany.dearlove@meduniwien.ac.at, andreas.bergthaler@meduniwien.ac.at

Data update version: v04

Analysis last run: 2024-04-17

# Aims

- Investigate the concordance of pango lineage classification between
  the GenBank and Viridian assemblies
- Identify and characterise indel changes between assemblies

# Results

There are n=2,205 pango lineages identified in the Viridian dataset,
and n=2,214 in the Genbank dataset. There are n=0 (0%) sequences
classified as unassigned in the Viridian assemblies versus n=81 (0.00%)
in the Genbank assemblies.

In the sections below, we first consider mismatches at the variant
level, and then look more closely at the lineage level.

## Variant level mismatches

### Overview

The plot below compares the classification of the Viridian and
Genbank assemblies at the variant level, which were split out by WHO
name, recombinant, unassigned lineage, or other. In general the Variant
level calls are consistent - only n=287 (0.01%) samples have different
variants called from the Genbank and Viridian assemblies.

### Association with other available metadata

#### By technology

#### By collection date

#### Country

## Lineage level

### Overall

Pango lineages are also generally consistent: only n=29,475 (0.98%)
samples have different lineages called from the Genbank and Viridian
assemblies.

The heatmaps below show an overview of how the Viridian (columns) and
Genbank (rows) assemblies were classified at the lineage level, coloured
by a) raw count and b) scaled by the number of Viridian sequences
classified as that lineage. Both axes are ordered by unaliased pango
lineage, thus the boxes of clustering yellow broadly align with
within-variant variation.

#### Raw count

#### Scaled to Viridian call

### Lineage relationships

For samples where the lineage was assigned differently between
assemblies, the relationship between the two lineages was defined
as:

- parent-child - i.e. B.1.1.7 and Q.1
- sibling - i.e. Q.1 and Q.2
- same variant - i.e. BA.2.13.1 and BA.5.1
- more distant - i.e. B.1.1.1 and BA.1
- one unassigned - if one of the assemblies could not be
  designated.

Most mismatches were at the parent-child level (77.17%), with the
Viridian assembly the child (i.e. more accurately designated) in 60.35%
of those.

#### All

#### Mis-matched only

### Impact of Ns

- n=1,604,389 (53.37%) of Genbank assemblies have no Ns, compared
  to n=1,197,638 (39.84%) of Viridian assemblies.
- n=1,246,558 (41.46%) of samples have same number of Ns in both
  assemblies (i.e. lie along y=x in the scatterplots below)

  - n=279,210 (9.29%) have more Ns in the Genbank assembly
  - n=1,480,639 (49.25%) of samples have more Ns in the Viridian
    assembly.

#### Scatterplot

#### Scatterplot by relationship

#### Scatterplot, unassigned lineage only

### Association with other available metadata

#### By technology

##### Match/mis-match

##### Lineage relationship

##### Lineage relationship (detail)

#### By collection date

##### Match/mis-match

##### Lineage relationship

##### Lineage relationship (detail)

#### Country

##### Match/mis-match

##### Lineage relationship

##### Lineage relationship (detail)

## Indels for consistent variant calls

Indels were extracted from `all_indels.by_run.json` for
samples with matching Viridian and GenBank variant-level call (or
BA-lineage call for Omicron). Scatterplots show for each indel the
percentage of assemblies it is identified in (with filter passed) by
Viridian (x-axis) versus GenBank (y-axis); indels are labeled with site
and nucleotide change if there is >5% difference between Viridian and
Genbank.

Tables give information on all indels identified in a variant,
including the amino acid changes predicted from the nucleotides using
`VariantAnnotation::predictCoding`.

The number of consensus deletions for variants (including all
sublineages) were extracted from Cornelius Roemer’s consensus .json file
on Github for
reference, however note this does not appear to contain insertions.

### Alpha

- There are 1,801 different indels identified (filter passed) in
  253,797 sequences.
- n=864 (50.38%) indels are found only in a single sample.
- There are 372 indels identified by only Viridian (of which n=307 are
  found in only one sample), and 56 by only Genbank (of which n=45 are
  singleton).
- According to the consensus sequence, we expect up to 4 deletions in
  Alpha and sublineages.
- We find 4 indels in both pipelines for at least 90% of samples and 4
  indels in at least 50% of samples.
- 41.69% of indels are not in frame (length not a multiple of 3).

### Beta

- There are 36 different indels identified (filter passed) in 848
  sequences.
- n=13 (36.11%) indels are found only in a single sample.
- There are 2 indels identified by only Viridian (of which n=2 are
  found in only one sample), and 0 by only Genbank (of which n=0 are
  singleton).
- According to the consensus sequence, we expect up to 2 deletions in
  Beta and sublineages.
- We find 2 indels in both pipelines for at least 90% of samples and 2
  indels in at least 50% of samples.
- 63.89% of indels are not in frame (length not a multiple of 3).

### Delta

- There are 6,821 different indels identified (filter passed) in
  1,218,875 sequences.
- n=3,313 (52.92%) indels are found only in a single sample.
- There are 1,714 indels identified by only Viridian (of which n=1,343
  are found in only one sample), and 374 by only Genbank (of which n=298
  are singleton).
- According to the consensus sequence, we expect up to 7 deletions in
  Delta and sublineages.
- We find 3 indels in both pipelines for at least 90% of samples and 3
  indels in at least 50% of samples.
- 40.16% of indels are not in frame (length not a multiple of 3).

### Epsilon

- There are 135 different indels identified (filter passed) in 6,137
  sequences.
- n=85 (63.91%) indels are found only in a single sample.
- There are 38 indels identified by only Viridian (of which n=29 are
  found in only one sample), and 2 by only Genbank (of which n=1 are
  singleton).
- According to the consensus sequence, we expect up to 0 deletions in
  Epsilon and sublineages.
- We find 0 indels in both pipelines for at least 90% of samples and 0
  indels in at least 50% of samples.
- 51.88% of indels are not in frame (length not a multiple of 3).

### Eta

- There are 25 different indels identified (filter passed) in 477
  sequences.
- n=7 (29.17%) indels are found only in a single sample.
- There are 0 indels identified by only Viridian (of which n=0 are
  found in only one sample), and 1 by only Genbank (of which n=0 are
  singleton).
- According to the consensus sequence, we expect up to 5 deletions in
  Eta and sublineages.
- We find 6 indels in both pipelines for at least 90% of samples and 6
  indels in at least 50% of samples.
- 54.17% of indels are not in frame (length not a multiple of 3).

### Gamma

- There are 81 different indels identified (filter passed) in 2,893
  sequences.
- n=41 (55.41%) indels are found only in a single sample.
- There are 13 indels identified by only Viridian (of which n=9 are
  found in only one sample), and 4 by only Genbank (of which n=3 are
  singleton).
- According to the consensus sequence, we expect up to 2 deletions in
  Gamma and sublineages.
- We find 2 indels in both pipelines for at least 90% of samples and 2
  indels in at least 50% of samples.
- 48.65% of indels are not in frame (length not a multiple of 3).

### Iota

- There are 58 different indels identified (filter passed) in 2,629
  sequences.
- n=27 (50%) indels are found only in a single sample.
- There are 8 indels identified by only Viridian (of which n=8 are
  found in only one sample), and 2 by only Genbank (of which n=2 are
  singleton).
- According to the consensus sequence, we expect up to 2 deletions in
  Iota and sublineages.
- We find 2 indels in both pipelines for at least 90% of samples and 2
  indels in at least 50% of samples.
- 57.41% of indels are not in frame (length not a multiple of 3).

### Kappa

- There are 9 different indels identified (filter passed) in 234
  sequences.
- n=6 (66.67%) indels are found only in a single sample.
- There are 1 indels identified by only Viridian (of which n=1 are
  found in only one sample), and 0 by only Genbank (of which n=0 are
  singleton).
- According to the consensus sequence, we expect up to 0 deletions in
  Kappa and sublineages.
- We find 1 indels in both pipelines for at least 90% of samples and 1
  indels in at least 50% of samples.
- 44.44% of indels are not in frame (length not a multiple of 3).

### Lambda

- There are 16 different indels identified (filter passed) in 107
  sequences.
- n=9 (60%) indels are found only in a single sample.
- There are 4 indels identified by only Viridian (of which n=4 are
  found in only one sample), and 0 by only Genbank (of which n=0 are
  singleton).
- According to the consensus sequence, we expect up to 2 deletions in
  Lambda and sublineages.
- We find 1 indels in both pipelines for at least 90% of samples and 2
  indels in at least 50% of samples.
- 46.67% of indels are not in frame (length not a multiple of 3).

### Mu

- There are 36 different indels identified (filter passed) in 503
  sequences.
- n=18 (52.94%) indels are found only in a single sample.
- There are 3 indels identified by only Viridian (of which n=3 are
  found in only one sample), and 1 by only Genbank (of which n=1 are
  singleton).
- According to the consensus sequence, we expect up to 1 deletions in
  Mu and sublineages.
- We find 1 indels in both pipelines for at least 90% of samples and 2
  indels in at least 50% of samples.
- 52.94% of indels are not in frame (length not a multiple of 3).

### Theta

- There are 6 different indels identified (filter passed) in 6
  sequences.
- n=1 (16.67%) indels are found only in a single sample.
- There are 1 indels identified by only Viridian (of which n=0 are
  found in only one sample), and 0 by only Genbank (of which n=0 are
  singleton).
- According to the consensus sequence, we expect up to 2 deletions in
  Theta and sublineages.
- We find 1 indels in both pipelines for at least 90% of samples and 3
  indels in at least 50% of samples.
- 83.33% of indels are not in frame (length not a multiple of 3).

### Zeta

- There are 3 different indels identified (filter passed) in 89
  sequences.
- n=2 (66.67%) indels are found only in a single sample.
- There are 1 indels identified by only Viridian (of which n=1 are
  found in only one sample), and 0 by only Genbank (of which n=0 are
  singleton).
- According to the consensus sequence, we expect up to 0 deletions in
  Zeta and sublineages.
- We find 0 indels in both pipelines for at least 90% of samples and 0
  indels in at least 50% of samples.
- 33.33% of indels are not in frame (length not a multiple of 3).

### Omicron

#### BA.1

- There are 5,296 different indels identified in 659,241
  sequences.
- According to the consensus sequence, we expect up to 6 deletions in
  BA.1 and sublineages.
- n=2,892 (59.63%) indels are found only in a single sample.
- There are 1,808 indels identified by only Viridian (of which n=1,406
  are found in only one sample), and 299 by only Genbank (of which n=248
  are singleton).
- We find 6 indels in both pipelines for at least 90% of samples and 7
  indels in at least 50% of samples.
- 37.18% of indels are not in frame (length not a multiple of 3).

#### BA.2

- There are 3,195 different indels identified in 437,496
  sequences.
- According to the consensus sequence, we expect up to 9 deletions in
  BA.2 and sublineages.
- n=1,682 (56.22%) indels are found only in a single sample.
- There are 878 indels identified by only Viridian (of which n=695 are
  found in only one sample), and 108 by only Genbank (of which n=88 are
  singleton).
- We find 4 indels in both pipelines for at least 90% of samples and 4
  indels in at least 50% of samples.
- 41.64% of indels are not in frame (length not a multiple of 3).

#### BA.3

- There are 12 different indels identified in 44 sequences.
- According to the consensus sequence, we expect up to 6 deletions in
  BA.3 and sublineages.
- n=6 (50%) indels are found only in a single sample.
- There are 0 indels identified by only Viridian (of which n=0 are
  found in only one sample), and 0 by only Genbank (of which n=0 are
  singleton).
- We find 6 indels in both pipelines for at least 90% of samples and 6
  indels in at least 50% of samples.
- 75% of indels are not in frame (length not a multiple of 3).

#### BA.4

- There are 587 different indels identified in 30,218 sequences.
- According to the consensus sequence, we expect up to 6 deletions in
  BA.4 and sublineages.
- n=331 (59.11%) indels are found only in a single sample.
- There are 93 indels identified by only Viridian (of which n=77 are
  found in only one sample), and 23 by only Genbank (of which n=20 are
  singleton).
- We find 6 indels in both pipelines for at least 90% of samples and 6
  indels in at least 50% of samples.
- 49.29% of indels are not in frame (length not a multiple of 3).

#### BA.5

- There are 2,183 different indels identified in 212,624
  sequences.
- According to the consensus sequence, we expect up to 8 deletions in
  BA.5 and sublineages.
- n=1,101 (53.63%) indels are found only in a single sample.
- There are 472 indels identified by only Viridian (of which n=380 are
  found in only one sample), and 103 by only Genbank (of which n=79 are
  singleton).
- We find 4 indels in both pipelines for at least 90% of samples and 5
  indels in at least 50% of samples.
- 46.76% of indels are not in frame (length not a multiple of 3).

### Indels for mis-matched variant calls

There are 206 sequences where a WHO variant has been assigned to both
the Viridian and Genbank assemblies but does not match. Repeating the
previous indel analysis summarised across all the sequences with variant
mismatch, we see a subset of indels found in more Viridian assemblies
than Genbank (left plot). These indels also overlap with the Variant
consensus indels from WHO variants (highlighted in red, defined as found
in >50% samples by both Viridian and Genbank in previous variant
analysis). We also see that Genbank assemblies have fewer indels called
per sample (right plot; dashed lines give medians).

To investigate further, we considered all pairs of Viridian-Genbank
WHO variant mismatches. For each Variant consensus indel, we compared
the number of samples where it was not identified (left of black line)
to that where it was (right of black line) using Viridian (blue) and
Genbank (red). The purple bar overlap shows where the presence/absence
is consistent between the two assemblies. The WHO variants in which the
indel is consensus are listed under the site identifier.

The biggest discrepancies are seen between Delta and Omicron
consensus indels, where variant-defining indel presence/absence aligns
with the Variant call.
